# The magnitude of selection on growth varies among years and increases under warming conditions in a subarctic seabird

**DOI:** 10.1093/evlett/qrad001

**Published:** 2023-02-28

**Authors:** Drew Sauve, Anne Charmantier, Scott A Hatch, Vicki L Friesen

**Affiliations:** Department of Biology, Queen’s University, Kingston, Ontario, Canada; CEFE, Univ Montpellier, CNRS, EPHE, IRD, Montpellier, France; Institute for Seabird Research and Conservation, Anchorage, AK, United States; Department of Biology, Queen’s University, Kingston, Ontario, Canada

**Keywords:** adaptation, climate change, development, natural selection, ontogeny, sibling competition

## Abstract

Because of ongoing rapid climate change, many ecosystems are becoming both warmer and more variable, and these changes are likely to alter the magnitude and variability of natural selection acting on wild populations. Critically, changes and fluctuations in selection can impact both population demography and evolutionary change. Therefore, predicting the impacts of climate change depends on understanding the magnitude and variation in selection on traits across different life stages and environments. Long-term experiments in wild settings are a great opportunity to determine the impact of environmental conditions on selection. Here we examined variability in the strength of selection on size traits of nestling black-legged kittiwakes (*Rissa tridactyla*) in a 25-year study including a food supplementation experiment on Middleton Island in the Gulf of Alaska. Using mixed effect models, we examined the annual variability of stage-specific and resource-specific selection gradients across 25 years. We found that (a) larger and heavier hatchlings were the most likely to survive during early ontogeny, (b) non-food supplemented younger nestlings in a brood experienced the strongest selection, and (c) warmer conditions increased the magnitude of selection on nestling mass and affected non-food supplemented and second-hatched nestlings the most. Our results suggested that variable resource dynamics likely caused some of the changes in selection from year to year and that warming conditions increased the strength of selection on subarctic seabird growth. However, our experimental manipulation revealed that local environmental heterogeneity could buffer the selection expected from broader climatic changes. Consequently, understanding the interactive effects of local conditions and general changes in climate seems likely to improve our ability to predict future selection gradients.

## Introduction

A major difficulty in predicting adaptative responses to human-altered environments is accounting for and understanding the drivers of variation in selection. The environment varies across time and space and these fluctuations result in variable selection for wild populations (Chevin *et al.*, 2015; Endler, 1986). In theory, fluctuations in selection could impact genetic variation, phenotypic plasticity, and the interplay of evolution and demography (Bürger, 1999; Gavrilets & Scheiner, 1993; Lande & Shannon, 1996; Via & Lande, 1985). However, determining the importance of variation in selection depends on empirically documenting patterns of selection in wild populations (Chevin *et al.*, 2015; Gamelon *et al.*, 2018; Le Vaillant *et al.*, 2021).

Environmental heterogeneity could alter how changes in selection driven by climate change impact a population (Corsini *et al.*, 2021; Garant *et al.*, 2007). Under low-resource conditions, larger individuals might be better able to persist longer without food and outcompete other individuals for limited food availability, which would translate into increased selection on nestling body mass (Mock & Parker, 1997; Peters, 1983). Because warmer conditions are expected to decrease food availability in many habitats (e.g., through phenological mismatch or decreased prey abundance) we might expect to see stronger selection for larger individuals in warm conditions.

To predict the changes in selection under climate change, we need to understand how it operates across life stages and whether selection changes in the same way across life stages with changing environmental conditions. Characterizing selection across ontogeny can provide insights into when the direction or strength of selection might change across life stages (Price & Grant, 1984). Strong selective events early in life might shape the within-generation distribution of phenotypes resulting in altered survival and reproductive output relative to a population not experiencing early life selection (Bell *et al.*, 2021). Further, determining the direction and strength of selection on early-life traits is a necessary first step to determining whether plastic responses are adaptive.

Here we examine the form and strength of selection on size traits in nestling black-legged kittiwakes (*Rissa tridactyla*) across ontogeny, with an experimental comparison between non-food supplemented and food supplemented nestlings. Because selection can act on multiple traits simultaneously (Lande & Arnold, 1983), we measured selection gradients for two traits we hypothesized to be important for nestling survival, mass, and wing length. Black-legged kittiwakes are long-lived (life expectancy ~13 years; Hatch *et al.*, 2020) pelagic gulls that breed annually on elevated ledges (e.g., cliff or artificial ledges). Kittiwakes on Middleton Island spend the nonbreeding season at variable distances from the breeding colony along the North–East Pacific and food supplemented birds remain closer to the colony and return earlier (Whelan *et al.*, 2020). Middleton kittiwakes breed in the early spring (April–May) and lay one to three egg clutches. Of all nests with eggs on Middleton Island, one, two, and three egg clutches correspond to ~10%, 88%, and 1% of non-food supplemented nests and ~4%, 91%, and 5% of food supplemented nests. The majority of nestlings hatch in June and July and fledge in July, August, and early September. Because kittiwakes often share a nest with a sibling, the survival of a nestling can depend on its ability to outcompete its sibling (Merkling *et al.*, 2014; White *et al.*, 2010). Hence the presence and size of a sibling, might shape the selection on nestling growth. We addressed three questions: (a) What is the strength and form of selection acting on nestling size traits across ontogeny, food environments, and nestling rank? (b) Does interannual variation in selection differ among ontogenic stages, food supplementation, and nestling rank? (c) If selection varies in magnitude or direction across years, is it predicted by environmental conditions that we expect to change under climate change? Generally, we expected that larger nestlings would be more likely to survive, and selection would be strongest early in ontogeny and for the second-hatched nestling in a brood. We expected higher variability in selection in non-food supplemented nestlings and higher variability in selection for second-hatched nestlings, as the competition faced by non-food supplemented, and second-hatched nestlings is likely to be more dependent on food conditions (Braun & Hunt, 1983; Hatch, 2013; Sauve *et al.*, 2021; White *et al.*, 2010). Finally, we expected that warmer sea-surface temperatures would predict years with stronger selection on nestling size. Warmer sea-surface temperatures result in poor foraging conditions for kittiwakes, which is expected to increase sibling competition and potentially amplify the competitive advantage of being large (Braun & Hunt, 1983; Gill & Hatch, 2002; Hatch, 2013; Merkling *et al.*, 2014). Foraging conditions for kittiwakes appear to be impacted by environmental conditions both two years prior to and during the breeding season (Sauve *et al.*, 2021; Whelan *et al.*, 2022). The main prey fishes of kittiwakes take about two years to mature so it is thought that environmental effects two years prior to a breeding season might impact prey availability in any given year (Doyle *et al.*, 2019; Whelan *et al.*, 2022). Here we evaluate both time periods as potential predictors of selection on nestling size traits.

## Methods

### Black-legged kittiwake colony and food supplementation experiment

We used 25 years (1996–2021) of data from a colony of black-legged kittiwakes on Middleton Island (Gulf of Alaska; 59°26’N, 146°20’W) where kittiwakes nest on an abandoned radar tower (Gill & Hatch, 2002). The tower is a 12-walled polygon where artificial nest sites were created on the upper storeys, allowing observation through one-way glass windows from inside the tower. Each year, research teams provided a subset of the nesting pairs with capelin *Mallotus villosus* ad libitum through a polyvinyl chloride tube at their nest site three times a day from May to mid-August (further details in Gill & Hatch, 2002). The primary prey of kittiwakes during colder breeding seasons was capelin, but in warm years kittiwakes foraged on a higher proportion of Pacific herring *Clupea pallasii*, invertebrates, myctophids Myctophidae, sable-fish *Anopoploma fimbria*, salmon *Oncorhynchus,* and sand lance *Ammodytes hexapterus* (Hatch, 2013). The same nesting sites were studied each year but parental pairs at fed sites changed because of death or competition for sites. Nests were checked twice daily (at 9:00 and 18:00 hr) throughout the season to record laying and hatching. Within a brood, eggs hatch an average of 1.64 days apart (Merkling *et al.*, 2014), and so first-hatched and second-hatched nestlings were distinguished with different colors of a nontoxic marker on their head to distinguish nestling rank (first-hatched marked with red and second-hatched marked with blue) until banded at ~5 days of age. Every 5 days from hatching to 40 days (i.e., close to fledging) mass was weighed to the nearest 0.1 g using an electronic scale and wing length (“size”) was measured to the nearest 1 mm using a wing ruler. Note that body condition (residuals of body mass regressed on wing length) was highly correlated to body mass (e.g., age 0 correlation = 0.96 [0.95, 0.96] and age 40 correlation = 0.98 [0.97, 0.99]) so we opted to use body mass and wing length in our analysis. Several experiments have been conducted on the nests previously (e.g., Merkling *et al.*, 2014, 2016), so we excluded data from nestlings that were experimentally manipulated (~9.1% of breeding attempts excluded, beyond food supplementation).

### Statistical analyses

Our goal was to examine viability selection on mass and size throughout ontogeny, so our response variable was always survival to the next age class and our predictor(s) were the traits measured at the current age class. We evaluated our different fitness functions (below) for traits measured 9 times throughout the growth period (Ages 0 [hatching day], 5, 10, 15, 20, 25, 30, 35, 40). In our fitness functions we evaluated body mass and wing length as predictors of survival and these traits were standardized within each age class by subtracting the mean trait value and dividing by the standard deviation. Depending on the fitness function we ran linear or non-linear generalized mixed models. All models of survival were run with a binomial error distribution and “logit” link function using the R package “brms” in R version 4.1.3 (Bürkner, 2017; R Core Team, 2021). Selection estimates were back transformed to data scale following Janzen and Stern (1998) and de Villemereuil *et al.* (2020).

### Fitness functions

Following recent work examining fluctuating selection (Chevin *et al.*, 2015; de Villemereuil *et al.*, 2020), we compared different fitness function shapes for each selective period: (a) a flat fitness function where survival is independent of mass or wing length, (b) a straight line where survival probability changes monotonically with mass or wing length, (c) a flat two-dimensional surface where survival changes monotonically as a function of mass *and* wing length, (d) a Gaussian fitness function where survival probability is optimized at some value of mass or wing length, and (e) a bivariate Gaussian landscape where survival probability is optimized as a function of mass *and* wing length. See Supplementary File for the function equations. We used a leave-one-out information criterion (LOOIC) derived from approximate cross-validation using Pareto-smoothed importance sampling to compare and evaluate the predictive performance of each fitness function (Vehtari *et al.*, 2017). The lowest LOOIC value was considered the best model and any model within 5 LOOIC values of the best LOOIC model was considered as a top model. For each of our fitness functions we estimated the parameters of each function at each food treatment and hatching order grouping using fixed effects. Additionally, because differences among ages in standardized selection gradients could be due to the measurement of selection at different points along a single non-linear selection function acting on mass independent of age (e.g., Hunter *et al.*, 2018), we estimated linear selection gradients on absolute non-standardized mass and wing length for each age category (fitness function 3 above). Differences among age-specific selection gradients measured for absolute mass and wing length will indicate whether our selection functions are indeed varying among age classes or are the result of our age categories sampling different segments of the same fitness function. While the above functions link theoretical models of selection with our empirical data, we also wanted to examine a less-constrained shape for the viability fitness function. So, we also modeled survival as a smooth function of standardized age-specific mass and wing length using a general additive model (GAM) and the default regression spline basis. We compared these GAM models to the fitness functions described above to detect potentially unexpected patterns that would go unnoticed in the abovementioned models.

### Variability of selection

For each selective episode, if our evaluation of fitness functions indicated that the monotonic or optimum fitness models best predicted survival, we allowed the fitness function to vary annually. Because we aimed to determine if different treatments or nestling ranks affected the variability of selection, we estimated the annual variance in our fitness function parameters for each experimental and rank grouping. If our best model of selection was a line or plane (fitness function shapes 2 and 3) we allowed the slope(s) to vary among years and if our best model of selection was an optimum function (shapes 4 and 5) we allowed the optimum(s) and maximum fitness parameters to vary among years. We did not allow the width of our optimal fitness functions to vary among years because our sample sizes were unlikely to provide sufficient power to estimate fluctuations in the fitness function width (de Villemereuil *et al.*, 2020). We first allowed our selection function to vary among years for all nestling rank and treatment groups (synchronous annual variation in selection among treatment and rank groups) and then allowed our selection function to vary differently for each nestling rank and treatment group (heterogeneous variation in selection among treatment and rank groups). We compared these two parameterizations using LOOIC to determine support for synchronous or heterogeneous variability of selection among rank and treatment groups (Vehtari *et al.*, 2017).

### Environmental correlates of selection

If our top model indicated that selection varied among years, we evaluated whether selection parameters correlated with four environmental variables: Pacific Decadal Oscillation (PDO) during the breeding season; minimum air temperature during the breeding season; average sea-surface temperature during the breeding season (previously shown to correlate with nestling growth parameters and annual fecundity in the population; Hatch, 2013; Sauve *et al.*, 2022); and PDO two years prior to breeding (previously shown to predict the laying date of kittiwakes at Middleton; Whelan *et al.*, 2022). We ran eight models and compared their LOOIC values to each other and to our top model of variation in selection without an environmental effect. Each model only contained one of the environmental variables described above, and for each environmental variable, we ran two models: one with the environmental variable as a fixed effect that impacts selection on all kittiwake ranks and treatments in the same way (no interaction between variable and our rank and treatment groups); and one with the environmental variable as a fixed effect that impacts selection parameters differently depending on a nestling’s rank and treatment (an interaction between environmental variable, nestling rank, and treatment).

## Results

### Overall patterns of selection

**Figure 1.**
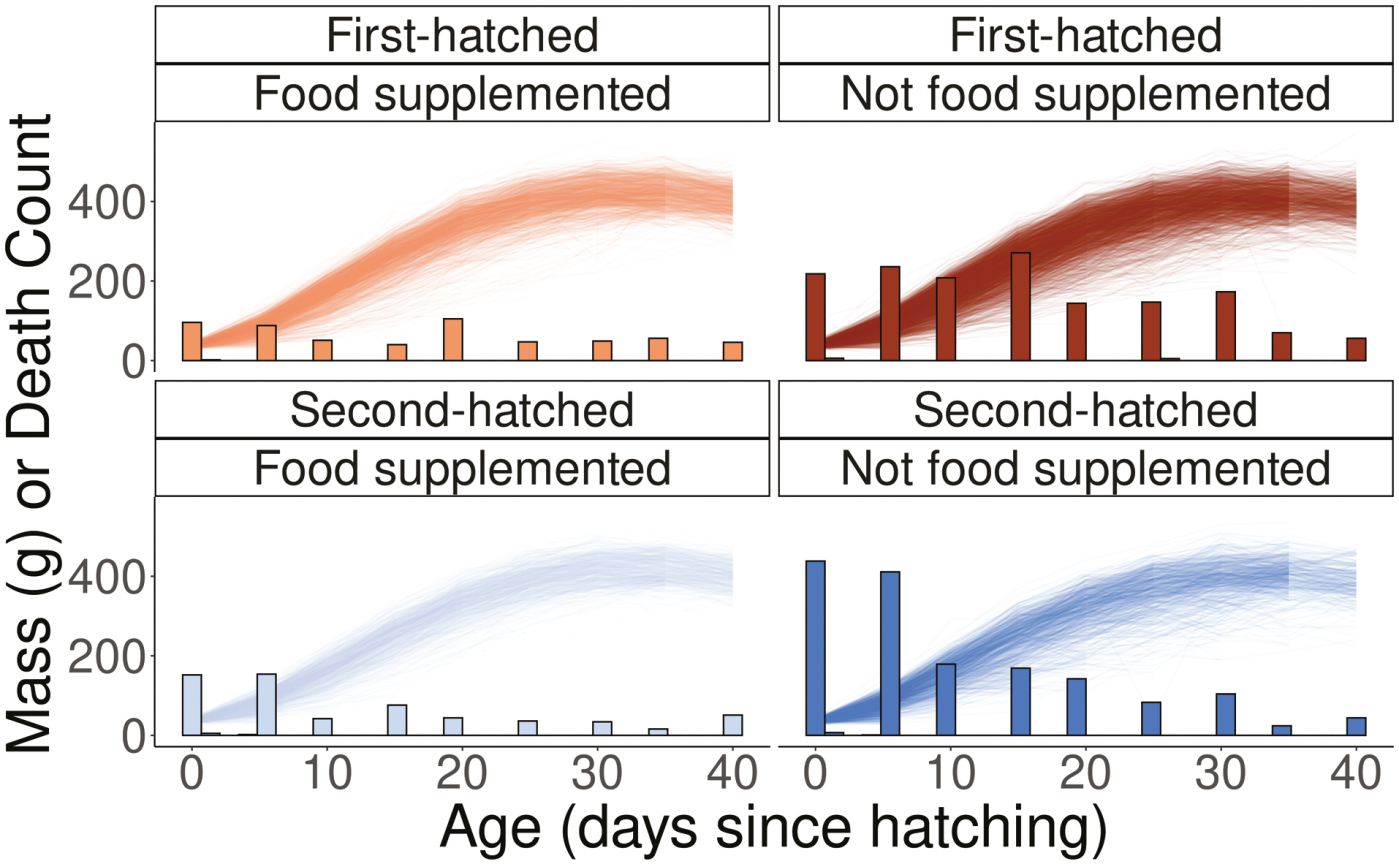
Death counts and individual nestling mass measurements at each age step considered in this study. Bars indicate the number of nestlings that died between one mass measurement and the next. There is only one y-axis because the left y-axis is either the mass of individuals in grams or the count of individuals that did not survive to the next measurement age. Thin lines indicate the growth mass measurements taken for each age and each individual. Bars and lines grouped by nestling rank and food treatment groups.

**Figure 2.**
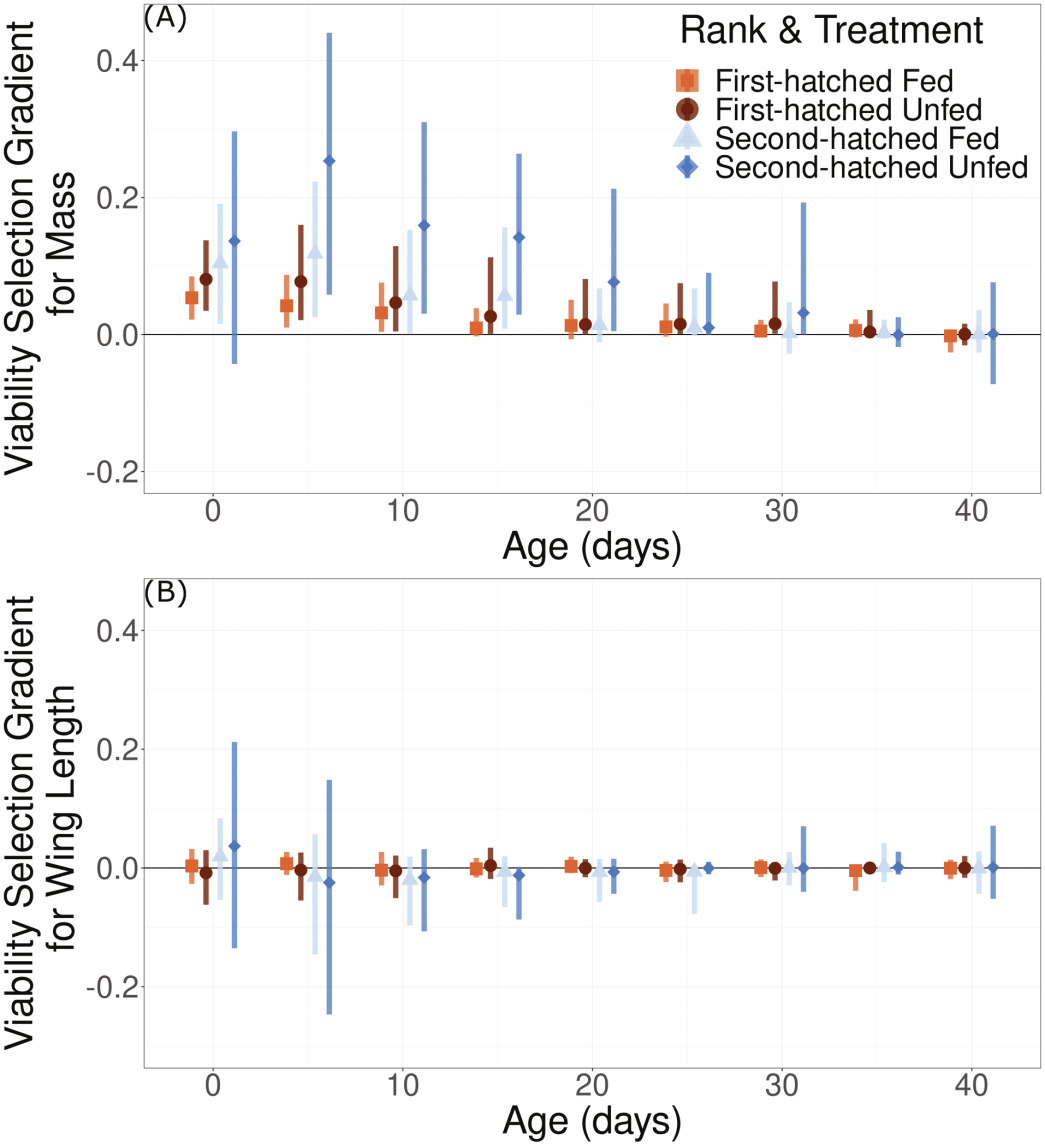
Viability selection gradients for (A) mass and (B) wing length. Selection gradients are transformed from logistic regression to the data scale following Janzen & Stern (1998) and de Villemereuil *et al.* (2020). Points and 95% confidence intervals are grouped by nestling rank and food treatment groups. Squares indicate first-hatched food supplemented nestlings, circles indicate first-hatched non-food supplemented nestlings, triangles indicate second-hatched food supplemented nestlings, diamonds indicate second-hatched non-food supplemented nestlings.

In all food treatment and nestling rank groups the highest nestling death counts occurred in the first 20 days after hatching, and deaths were most pronounced in the non-food supplemented nestlings (Figure 1; Table 1). Heavier nestlings were more likely to survive to the next size measurement (Figure 2A) and wing length did not predict survival throughout the nestling period (Figure 2B). Selection gradients for mass were strongest during early ontogeny for non-food supplemented compared to food supplemented nestlings (Figure 2A), and for second-hatched compared to first-hatched nestlings. However, differences in selection among groups disappeared as nestlings aged, and did not exist for selection gradients on wing length (Figure 2B). Selection gradients measured for absolute mass also indicated selection was the strongest during early ontogeny, but indicated a more rapid drop off in selection strength with age (Supplementary Figure 1). The top fitness function for each selective period always included mass, but during early ontogeny our top fitness function tended to include wing length (ages 0, 10, and 15, but not 5; Supplementary Material; Supplementary Figure 2; Supplementary Tables 1–9). Our best (lowest LOOIC) fitness function of compared functions at hatching was a bivariate Gaussian function of mass and wing length, while the best functions at 10 and 15 days of age were flat planar functions of mass and wing length (Supplementary Figure 2). The best fitness functions at every other age (5, 20, 25, 30, 35, and 40) were linear functions of age-specific masses (Supplementary Figure 2). GAMs indicated that in most cases survival probability plateaued as mass increased, but with larger uncertainty in survival probability at the heaviest masses at age 0, 5, and 40 (Supplementary Figure 3). GAMs indicated generally flat fitness functions for wing length, but with large uncertainties of survival probability at short- and long-wing lengths (Supplementary Figure 4).

**Table 1.** Sample sizes of nestlings at each age class by hatching order and food treatment with trait measurements.

Age	Non-food supplemented		Food supplemented	
	First-hatched	Second-hatched	First-hatched	Second-hatched
**0**	1,923	1,200	1,098	738
**5**	1,728	786	1,004	575
**10**	1,539	573	954	511
**15**	1,511	531	964	505
**20**	1,416	480	950	490
**25**	1,461	463	910	463
**30**	1,270	422	878	458
**35**	1,176	391	849	439
**40**	668	224	671	349

### Variability of selection

All age-specific fitness functions that allowed annual variation in selection functions outperformed models with constant selection across years (Supplementary Tables 1–9), except the fitness function at age 30. In almost all cases half of the top selection models allowed the fitness function parameters to vary synchronously among years and half allowed fitness function parameters to vary differently among years for all hatching rank and food supplementation groups (Supplementary Tables 1–9).

### Environmental correlates of selection

**Figure 3.**
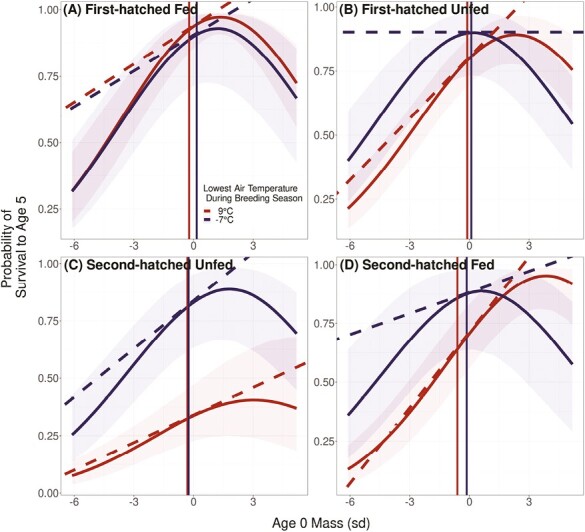
The association between the minimum air temperature during a breeding season and the fitness function from Age 0 to 5 with heterogeneous effects on each nestling rank and food treatment group. For first-hatched food supplemented (A), first-hatched non-food supplemented (B), first-hatched non-food supplemented (C), and second-hatched non-food supplemented (D) the conditional (holding wing-length constant) Gaussian fitness function of mass is displayed under the warmest minimum air temperature during the breeding season (9 °C; red) and minimum air temperature during the breeding season (−7 °C; dark blue). Solid curves lines indicate the Gaussian fitness function with associated 95% confidence intervals, while solid vertical lines indicate the average mass of each nestling group under warm or cold conditionals. Diagonal or horizontal dashed lines indicate the selection operating each nestling group under each temperature condition.

The only selective periods when models of survival were improved by the addition of environmental predictors were at ages 0 and 15 days. During all other selective periods a model without any environmental effects that *only* included annual fluctuations in the fitness function was the best or a top model (ΔLOOIC = 0 or ΔLOOIC <5; Supplementary Tables 20–37). During the selective periods when an environmental predictor improved the fitness function (ages 0 and 15) the lowest air temperature during the breeding season was the environmental predictor of selection parameters. We identified two top models for survival from age 0 to 5 that both suggested the lowest air temperature during the breeding season affected selection parameters, but one model suggested air temperature increased the optimal mass for all groups and the other suggested air temperature altered fitness function parameters differently for each nestling rank and treatment (Supplementary Table 29; Figure 3). The best and only top model for survival from age 15 to 20 suggested stronger selection on mass under warmer air temperature conditions (Supplementary Table 32). Additionally, for both selective periods (age 0–5 and age 15–20) survival was slightly higher for nestlings with shorter wing lengths under warmer conditions (Supplementary Tables 29 and 32).

## Discussion

Based on patterns of nestling survival in a long-term experimental study of black-legged kittiwakes, we found that (a) selection on nestling mass weakened with time after hatching and selection on mass in an average year was strongest for second-hatched and non-food supplemented nestlings, (b) interannual variation in the magnitude of selection was ubiquitous across the nestling growth period, and (c) when selection was strongest (the first 20 days after hatching) warmer conditions selected for heavier nestlings with shorter wings and colder conditions weakened the magnitude of selection.

### Selection on mass

Like many investigations of phenotypic selection on size traits we found evidence of selection favoring heavier individuals (Hajduk *et al.*, 2020; Kingsolver & Diamond, 2011). Kittiwakes exhibit facultative siblicide and larger nestlings might be better able to survive aggression from siblings (Braun & Hunt, 1983). Increased mass could also be advantageous because of an increased tolerance to stressful thermal environments or an ability to persist when food is scarce (Hone & Benton, 2005; Peters, 1983, p. 67–78). Whether the consistent (in direction) selection observed for all groups leads to evolutionary change in mass of nestlings will depend on whether there is an additive genetic covariation between nestling mass and survival or reproduction (Price *et al.*, 1988; e.g., Hajduk *et al.*, 2020). Evolutionary change will also depend on trade-offs between parental fitness and nestling traits (Willham, 1972). Understanding the evolution of nestling traits depends on understanding the drivers of fitness in parents and offspring and here we provide a detailed investigation of selection on offspring (Hadfield *et al.*, 2008; Thomson *et al.*, 2017).

We examined the early developmental stage of a generally long-lived seabird and found decreasing selection gradients with age. Like our study, investigations of blue tits (*Cyanistes caeruleus*) and southern elephant seals (*Mirounga leonine*), also found stronger selection for mass during early ontogeny vs. late ontogeny (Oosthuizen *et al.*, 2018; Thomson *et al.*, 2017). These empirical results follow expectations with senescence theory that traits expressed early in life will have a greater impact on fitness than traits expressed late in life (Williams, 1957). However, in some taxa (e.g., many plants) age might not be and good predictor of developmental stage. In these cases, selection strength and age could covary positively within a developmental stage (e.g., Caswell and Salguero-Gόmez, 2013; Roper *et al.*, 2021).

Because selection is often estimated for traits that are only expressed or measured later in life, total selection for body mass may frequently be underestimated. As some individuals do not survive to express traits later in life, selection measured at later stages does not account for selection against phenotypes that are counter selected early in life (Hadfield *et al.*, 2008). Depending on the phenotypic and additive genetic (co)variance of mass across early and late age classes—the distribution of phenotypes and breeding values for mass in adult birds could be shaped by early life selection in addition to the commonly documented selection for heavier masses during adult stages (Gomulkiewicz *et al.*, 2018; Hadfield, 2013; Thomson *et al.*, 2017). While we still need information on the additive genetic covariance of mass, wing length, and survival to make predictions of evolutionary change, measurements of phenotypic selection indicate that (a) any plastic mechanisms that result in heavier nestlings (at early life stages and under all our explored environmental conditions) are adaptive in terms of offspring viability and (b) the within generation change of phenotypes are shaped such that relatively heavier individuals outcompete lighter individuals early in life. Whether these within generation changes in phenotypes affect survival and reproductive output at later life stages warrants investigation (e.g., Bell *et al.*, 2021).

### Variation in the magnitude of selection

We found evidence for annual variation in selection across each growth episode. To date, studies of fluctuating selection have focused on phenological traits, and our study agrees with previously described patterns of selection on phenology in birds and mammals that suggests that selection tends to vary among years in magnitude but not direction (de Villemereuil *et al.*, 2020).

While we predicted among-year variation in selection to be greater for non-food supplemented and second-hatched nestlings, we did not detect differences in variability of selection among groups. We did however identify large confidence intervals for estimates of selection on mass in non-food supplemented and second-hatched nestlings (Figure 2). Large confidence intervals in selection parameters could suggest that selection on mass of second-hatched nestlings depends greatly on within breeding season variation. A recent study of snapdragons (*Antirrhinum majus* L.) indicates that natural selection can vary at very small spatial scales and broadly many investigations of adaptive phenotypic and genetic differences suggest that selection must sometimes vary at fine spatial scales (Marrot *et al.*, 2022; Richardson *et al.*, 2014). The microclimates of the radar tower on Middleton Island can vary greatly and selection could vary even across small spatial scales (Lacey, 2018). Further, we demonstrated that differences in resource environments experienced by nestlings might alter the strength of selection on mass (Figure 2). Therefore, any differences in resource environment because of parental traits like phenology or care are also potential causes of variation in selection experienced by second-hatched non-food supplemented nestlings (Sauve *et al.*, 2021).

### Environmental predictors of selection and implications for climate change

We evaluated several environmental variables as possible predictors of fitness function parameters. Lagged PDO, which influences breeding phenology, does not also predict the selective environment that nestlings will face. Instead, minimum air temperature during a breeding season is associated with stronger positive selection on mass at earlier ages. Interestingly, this variable was associated with slower growth and smaller sizes at fledging (Sauve *et al.*, 2021). An association between warmer air temperatures and smaller nestlings could suggest that nestlings might be plastically adjusting to a smaller size under the same conditions that select for larger nestlings (i.e., a maladaptive plastic response).

Warmer conditions seem likely to increase the strength of selection on mass and, possibly, wing length. This association between mass and survival contrasts with some predictions that climate change will favor smaller body size (Millien *et al.*, 2006; Teplitsky & Millien, 2014). But, the hypothesized mechanism driving selection for smaller body sizes is expected to arise from improved heat tolerance associated with small size (Salewski & Watt, 2017). We think that the main mechanism of selection acting here is competition among siblings, and kittiwake nestlings seem unlikely to be thermally challenged regularly as air temperatures do not exceed thermal neutral temperatures of 33–35 °C (Bech *et al.*, 1984). We hypothesize that in years of poor foraging conditions, heavier nestlings will be better able to survive periods of low food availability and increased aggression from siblings and neighbors (Hone & Benton, 2005). Across many taxa, heavier individuals are able to survive periods of scarcity and in many avian species, especially those with asynchronous hatching, sibling competition is thought to increase under poor food conditions (Mock & Parker, 1997; Peters, 1983, p. 24–43). Starvation itself may modulate increased aggression in nestlings, or parents may adjust food allocation or egg hormones affecting behavioral interactions in the nest (Groothuis *et al.*, 2005; Shizuka & Lyon, 2013). Our results suggest that in species with environmentally modulated sibling competition, food supplementation (e.g., bird feeders or human garbage) could affect rates of adaptation to warming climatic conditions.

## Conclusions

We conclude that selection for heavier black-legged kittiwakes during early ontogeny was constant in direction but increased in magnitude under warmer conditions. We confirm that in black-legged kittiwakes early ontogeny is a period of strong selection and that this could be true for other long-lived species that experience variable environmental conditions. The specific changes in strength of selection we detected were affected by the food and sibling environment a nestling experienced. Future research will further decompose the variation in nestling mass and survival into genetic, and environmental components to determine if evolutionary change is expected under warming conditions. Further, the implications of a within generation change toward larger masses should be investigated, and plastic responses in mass should be quantified to determine whether they are adaptive or not.

## Supplementary Material

qrad001_suppl_Supplementary_Material

## Data Availability

Data and code are available on figshare doi: 10.6084/m9.figshare.21743759.
