# Supplementary material for: The magnitude of selection on growth varies among years and increases under warming conditions in a subarctic seabird
Source: Evol Lett. 2023 Feb 28;8(1):56–63. doi: 10.1093/evlett/qrad001 (PMC10871900; doi:10.1093/evlett/qrad001)
Supplement: qrad001_suppl_Supplementary_Material [file qrad001_suppl_supplementary_material.pdf]

Table of Contents

Fitness Functions 4

Supplementary Table 1: ΔLOOIC values for fitness functions evaluated for survival from age 0 to age 5 as a function of mass and/or wing length........ 5

Supplementary Table 2: ΔLOOIC values for fitness functions evaluated for survival from age 5 to age 10 as a function of mass and/or wing length. 6

Supplementary Table 3: ΔLOOIC values for fitness functions evaluated for survival from age 10 to age 15 as a function of mass and/or wing length. 7

Supplementary Table 4: ΔLOOIC values for fitness functions evaluated for survival from age 15 to age 20 as a function of mass and/or wing length. 8

Supplementary Table 5: ΔLOOIC values for fitness functions evaluated for survival from age 20 to age 25 as a function of mass and/or wing length. 9

Supplementary Table 6: ΔLOOIC values for fitness functions evaluated for survival from age 25 to age 30 as a function of mass and/or wing length. 10

Supplementary Table 7: ΔLOOIC values for fitness functions evaluated for survival from age 30 to age 35 as a function of mass and/or wing length. 11

Supplementary Table 8: ΔLOOIC values for fitness functions evaluated for survival from age 35 to age 40 as a function of mass and/or wing length. 12

Supplementary Table 9: ΔLOOIC values for fitness functions evaluated for survival from age 40 to fledging as a function of mass and/or wing length. 13

Supplementary Table 10: Top fitness function for survival from age 0 to age 5 based on ΔLOOIC comparisons. 14

Supplementary Table 11: Top fitness function for survival from age 5 to age 10 based on ΔLOOIC comparisons. 15

Supplementary Table 12: Top fitness function for survival from age 10 to age 15 based on ΔLOOIC comparisons. 16

Supplementary Table 13: Top fitness function for survival from age 15 to age 20 based on ΔLOOIC comparisons. 17

Supplementary Table 14: Top fitness function for survival from age 20 to age 25 based on ΔLOOIC comparisons. 18

Supplementary Table 15: Top fitness function for survival from age 25 to age 30 based on ΔLOOIC comparisons. 19

Supplementary Table 16: Top fitness function for survival from age 30 to age 35 based on ΔLOOIC comparisons. 20

Supplementary Table 17: Top fitness function for survival from age 35 to age 40 based on ΔLOOIC comparisons. 21

Supplementary Table 18: Top fitness function for survival from age 40 to age fledging based on ΔLOOIC comparisons. 22

Supplementary Table 19: Among year standard deviation in fitness function parameters by selective period. 23
Supplementary Table 20: ΔLOOIC values for fitness functions evaluated for survival from age 0 to age 5 as a function of mass and/or wing length. 24
Supplementary Table 21: ΔLOOIC values for fitness functions evaluated for survival from age 5 to age 10 as a function of mass and/or wing length. 25
Supplementary Table 22: ΔLOOIC values for fitness functions evaluated for survival from age 10 to age 15 as a function of mass and/or wing length. 26
Supplementary Table 23: ΔLOOIC values for fitness functions evaluated for survival from age 15 to age 20 as a function of mass and/or wing length. 27
Supplementary Table 24: ΔLOOIC values for fitness functions evaluated for survival from age 20 to age 25 as a function of mass and/or wing length. 28
Supplementary Table 25: ΔLOOIC values for fitness functions evaluated for survival from age 25 to age 30 as a function of mass and/or wing length. 29
Supplementary Table 26: ΔLOOIC values for fitness functions evaluated for survival from age 30 to age 35 as a function of mass and/or wing length. 30
Supplementary Table 27: ΔLOOIC values for fitness functions evaluated for survival from age 35 to age 40 as a function of mass and/or wing length. 31
Supplementary Table 28: ΔLOOIC values for fitness functions evaluated for survival from age 0 to age 5 as a function of mass and/or wing length. 32
Supplementary Table 29: Top fitness function for survival from age 0 to age 5 based on ΔLOOIC comparisons. 33
Supplementary Table 30: Top fitness function for survival from age 5 to age 10 based on ΔLOOIC comparisons. 34
Supplementary Table 31: Top fitness function for survival from age 10 to age 15 based on ΔLOOIC comparisons. 35
Supplementary Table 32: Top fitness function for survival from age 15 to age 20 based on ΔLOOIC comparisons. 36
Supplementary Table 33: Top fitness function for survival from age 20 to age 25 based on ΔLOOIC comparisons. 37
Supplementary Table 34: Top fitness function for survival from age 25 to age 30 based on ΔLOOIC comparisons. 38
Supplementary Table 35: Top fitness function for survival from age 30 to age 35 based on ΔLOOIC comparisons. 39
Supplementary Table 36: Top fitness function for survival from age 35 to age 40 based on ΔLOOIC comparisons. 40
Supplementary Table 37: Top fitness function for survival from age 40 to fledging based on ΔLOOIC comparisons. 41
Supplemental Figure 1: Viability selection gradients for absolute A) mass and B) wing length. 42

Supplemental Figure 2: Summary of selection functions and variability of selection parameters for each selective period (Age in number of days on the X-axis). 43
Supplemental Figure 3: Generalized additive model predicted survival as a function of nestling age specific mass. 44
Supplemental Figure 4: Generalized additive model predicted survival as a function of nestling age specific wing length. 45
References 46

Fitness Functions

As noted in the main text we contrasted five different fitness function shapes at each survival stage. Following de Villemereuil *et al.*, (2020) we used a logit link function for our flat, linear, and planar fitness functions (eqs. 1a, 1b, & 1c) but used an exponential for the Gaussian or bivariate Gaussian fitness peak and a logit link for the W_{max} (maximum fitness) parameter (eqs 1d &1e). In each of the below equations the survival probability is modelled as a function of either one trait (mass or wing length; z). or two traits (mass & wing length; z_1 & z_2). The below equations correspond to the fitness functions described in the text

$$W(z) = logit\ (a) \tag{1a}$$

$$W(z) = logit\ (a + bz) \tag{1b}$$

$$W(z_1, z_2) = logit\ (a + b_1 z_1 + b_2 z_2) \tag{1c}$$

$$W(z) = logit\ (W_{max}) exp\left(-\frac{(z-\theta)^2}{2\omega^2}\right) \tag{1d}$$

$$W(z_1, z_2) = logit\ (W_{max}) exp\left[-\frac{\left(\frac{z_1-\theta_1}{{\omega_1}^2} - \frac{2\rho(z_1-\theta_1)(z_2-\theta_2)}{\omega_1\omega_2} + \frac{z_2-\theta_2}{{\omega_2}^2}\right)}{2(1-\rho^2)}\right] \tag{1e}$$

where a is the intercept (Eq. 1a, b, c), b is the selection differential (Eq. 1b) or gradient (Eq. 1c), W_{max} is the maximum fitness, θ is the optimal mass or wing length (Eqs. 1d, e), ω is the width of the fitness function (Eqs. 1d, e), and ρ is the correlation between mass and wing length (Eq. 1e).

Supplementary Table 1: ΔLOOIC values for fitness functions evaluated for survival from age 0 to age 5 as a function of mass and/or wing length. ΔLOOIC values are the differences between LOOIC values of the best model (lowest LOOIC value) and LOOIC value for a given model. Models with a ΔLOOIC <5 are bolded.

Survival Period	Model	ΔLOOIC
Survival from 0 to 5 Days	**Fluctuating Bivariate Gaussian**	**0**
	**Fluctuating Bivariate Gaussian by Group**	**1.8**
	Bivariate Gaussian	151.3
	Univariate Gaussian mass	160.1
	Directional mass	165.8
	Plane mass & wing	169.3
	Univariate Gaussian wing	337.7
	Directional wing	343.9
	Flat	355.3

Supplementary Table 2: ΔLOOIC values for fitness functions evaluated for survival from age 5 to age 10 as a function of mass and/or wing length. ΔLOOIC values are the differences between LOOIC values of the best model (lowest LOOIC value) and LOOIC value for a given model. Models with a ΔLOOIC <5 are bolded.

Survival Period	Model	ΔLOOIC
Survival from 5 to 10 Days	**Fluctuating Linear Mass by Group**	**0**
	**Fluctuating Linear Mass**	**2.3**
	**Fluctuating Plane mass & Wing by Group**	**4.7**
	**Fluctuating Plane mass & Wing**	**4.9**
	Directional mass	63.4
	Plane mass & wing	66.2
	Bivariate Gaussian	111.4
	Univariate Gaussian weight	144.7
	Univariate Gaussian wing	397.5
	Directional wing	360.9
	Flat	633.3

Supplementary Table 3: ΔLOOIC values for fitness functions evaluated for survival from age 10 to age 15 as a function of mass and/or wing length. ΔLOOIC values are the differences between LOOIC values of the best model (lowest LOOIC value) and LOOIC value for a given model. Models with a ΔLOOIC <5 are bolded.

Survival Period	Model	ΔLOOIC
Survival from 10 to 15 Days	**Fluctuating Bivariate Plane**	**0**
	**Fluctuating Linear Mass by Group**	**1.3**
	**Fluctuating Linear Mass**	**2.3**
	**Plane mass & wing**	**3.9**
	**Fluctuating Bivariate Plane by Group**	**4.2**
	Linear mass	9.9
	Bivariate Gaussian	56.1
	Univariate Gaussian weight	83.4
	Directional wing	143.9
	Univariate Gaussian wing	179.2
	Flat	312.3

Supplementary Table 4: ΔLOOIC values for fitness functions evaluated for survival from age 15 to age 20 as a function of mass and/or wing length. ΔLOOIC values are the differences between LOOIC values of the best model (lowest LOOIC value) and LOOIC value for a given model. Models with a ΔLOOIC <5 are bolded.

Survival Period	Model	ΔLOOIC
Survival from 15 to 20 Days	**Fluctuating Bivariate Plane**	**0**
	**Fluctuating Bivariate Plane by Group**	**3.5**
	Plane mass & wing	18.1
	Linear mass	25
	Bivariate Gaussian	63.4
	Univariate Gaussian weight	100.2
	Directional wing	150.2
	Univariate Gaussian wing	183.9
	Flat	329.9

Supplementary Table 5: ΔLOOIC values for fitness functions evaluated for survival from age 20 to age 25 as a function of mass and/or wing length. ΔLOOIC values are the differences between LOOIC values of the best model (lowest LOOIC value) and LOOIC value for a given model. Models with a ΔLOOIC <5 are bolded.

Survival Period	Model	ΔLOOIC
Survival from 20 to 25 Days	**Fluctuating Linear Mass**	**0**
	**Linear mass**	**1.9**
	**Fluctuating Bivariate Plane**	**3.6**
	**Fluctuating Linear Mass by Group**	**3.6**
	**Plane mass & wing**	**4**
	Bivariate Gaussian	11.4
	Fluctuating Bivariate Plane by Group	13.4
	Univariate Gaussian weight	25.3
	Directional wing	60.6
	Univariate Gaussian wing	70.7
	Flat	148.6

Supplementary Table 6: ΔLOOIC values for fitness functions evaluated for survival from age 25 to age 30 as a function of mass and/or wing length. ΔLOOIC values are the differences between LOOIC values of the best model (lowest LOOIC value) and LOOIC value for a given model. Models with a ΔLOOIC <5 are bolded.

Survival Period	Model	ΔLOOIC
Survival from 25 to 30 Days	**Fluctuating Linear Mass by Group**	**0**
	Fluctuating Linear Mass	5.5
	Linear mass	8.1
	Fluctuating Bivariate Plane	8.3
	Bivariate Plane	12.3
	Bivariate Gaussian	12.8
	Univariate Gaussian weight	26.7
	Directional wing	50.9
	Univariate Gaussian wing	61.5
	Flat	87.3

Supplementary Table 7: ΔLOOIC values for fitness functions evaluated for survival from age 30 to age 35 as a function of mass and/or wing length. ΔLOOIC values are the differences between LOOIC values of the best model (lowest LOOIC value) and LOOIC value for a given model. Models with a ΔLOOIC <5 are bolded.

Survival Period	Model	ΔLOOIC
Survival from 30 to 35 Days	**Fluctuating Linear Mass**	**0.0**
	Bivariate Gaussian	2.7
	Fluctuating Bivariate Plane	7.2
	Bivariate Plane	8
	Fluctuating Linear Mass by Group	9
	Univariate Gaussian weight	14.5
	Fluctuating Bivariate Plane by Group	21
	Univariate Gaussian wing	36.6
	Directional wing	51.4
	Flat	69.2

Supplementary Table 8: ΔLOOIC values for fitness functions evaluated for survival from age 35 to age 40 as a function of mass and/or wing length. ΔLOOIC values are the differences between LOOIC values of the best model (lowest LOOIC value) and LOOIC value for a given model. Models with a ΔLOOIC <5 are bolded.

Survival Period	Model	ΔLOOIC
Survival from 35 to 40 Days	**Fluctuating Linear Mass**	**0**
	**Fluctuating Linear Mass by Group**	**1**
	**Fluctuating Bivariate Plane**	**1.7**
	**Fluctuating Bivariate Plane by Group**	**2.6**
	Univariate Gaussian weight	5.1
	Linear mass	6.4
	Bivariate Plane	8
	Bivariate Gaussian	7.2
	Univariate Gaussian wing	13.3
	Flat	20.3
	Directional wing	21

Supplementary Table 9: ΔLOOIC values for fitness functions evaluated for survival from age 40 to fledging as a function of mass and/or wing length. ΔLOOIC values are the differences between LOOIC values of the best model (lowest LOOIC value) and LOOIC value for a given model. Models with a ΔLOOIC <5 are bolded.

Survival Period	Model	ΔLOOIC
Survival from 40 days to fledge	**Fluctuating Linear Mass by Group**	**0**
	**Fluctuating Linear Mass**	**0.4**
	Fluctuating Bivariate Plane	5.1
	Bivariate Gaussian	8.9
	Univariate Gaussian weight	9.3
	Flat	9.4
	Univariate Gaussian wing	10.8
	Directional wing	13.9
	Linear mass	17.5
	Bivariate Plane	22.3

Supplementary Table 10: Top fitness function for survival from age 0 to age 5 based on ΔLOOIC comparisons. For each model parameter the mean (Estimate), standard deviation (Est. Error), lower 95% credible interval (l-95% CI), and upper 95% credible interval (u-95% CI) are reported for the posterior distribution of that parameter. Rhat is an indicate of convergence of chains and if it is considerably greater than 1 the chains have not converged. The Bulk effective sampling size (Bulk_ESS) is a diagnostic for sampling efficiency of the bulk of the posterior and the tail effective sampling size (Tail_ESS) is a diagnostic for sampling efficiency of the tails of the posterior(see Bürkner, 2017; Vehtari *et al.*, 2017). Parameters under the "Population-Level" heading indicate fitness function parameters estimated across the entire dataset. Parameters under the "Group Level" heading indicate variation (standard deviation) in annual mean deviations from the population level parameters. The different parameters estimated are the maximum fitness Wmax, the trait specific optimum θ, the correlation between wing length and mass ρ, and the width of the fitness function for each trait ω. The suffices indicate the treatment and nestling rank level that a parameter was estimated at relative to the first-hatched food supplemented nestling group. For example, the optimal mass for a non-food supplemented first-hatched nestling would be sum of θ*_Mass_First-hatched_Fed* and θ_ *Mass_Treatmentunfed*. If there is no suffix for a parameter the same parameter was assumed for all food treatment and rank groupings.

**Age 0 Model of Selection**							
**Bivariate Gaussian Synchronous Fluctuations, Total observations = 4959**							
Group-Level Effects:							
Year	(Number	of	levels:	24)			
	Estimate	Est.Error	l-95% CI	u-95%CI	Rhat	Bulk_ESS	Tail_ESS
sd(Wmax)	0.64	0.43	0.04	1.64	1	1173	1347
sd(θ_Mass)	0.47	0.18	0.12	0.84	1.01	503	470
sd(θ_Wing)	3.11	0.8	1.82	4.92	1	1668	1989
Population-Level	Effects:						
	Estimate	Est.Error	l-95% CI	u-95%CI	Rhat	Bulk_ESS	Tail_ESS
Wmax	4.08	0.38	3.43	4.95	1	2956	1860
ρ_First-hatched_Fed	0.14	0.2	-0.28	0.51	1	1388	1526
ρ_Second-hatched	-0.08	0.28	-0.65	0.44	1	1578	1832
ρ_Treatmentunfed	-0.37	0.24	-0.84	0.1	1	1497	1384
ρ_Second-hatched:Treatmentunfed	-0.42	0.31	-0.94	0.25	1	1405	1748
θ_Mass_First-hatched_Fed	1.47	0.32	0.9	2.15	1	1514	1332
θ_ Mass_Second-hatched	1.17	0.37	0.36	1.85	1	1599	1323
θ_ Mass_Treatmentunfed	-0.02	0.36	-0.77	0.64	1	1301	1697
θ_ Mass_Second-hatched:Treatmentunfed	-3.49	0.83	-5.04	-1.86	1	2325	2195
θ_ Wing_First-hatched_Fed	0.16	1.6	-2.95	3.36	1	1185	1505
θ_Wing_Second-hatched	3.15	2.88	-2.88	8.67	1	1433	1731
θ_ Wing_Treatmentunfed	3.04	1.71	-0.12	6.78	1	1488	1882
θ_Wing_Second-hatched:Treatmentunfed	5.97	3.28	0.24	13.01	1	1478	1800
ω_Mass	5.08	0.51	4.23	6.2	1	1481	1602
ω_Wing	18.4	3.2	12.88	25.37	1	2427	2036

Supplementary Table 11: Top fitness function for survival from age 5 to age 10 based on ΔLOOIC comparisons. For each model parameter the mean (Estimate), standard deviation (Est. Error), lower 95% credible interval (l-95% CI), and upper 95% credible interval (u-95% CI) are reported for the posterior distribution of that parameter. Rhat is an indicate of convergence of chains and if it is considerably greater than 1 the chains have not converged. The Bulk effective sampling size (Bulk_ESS) is a diagnostic for sampling efficiency of the bulk of the posterior and the tail effective sampling size (Tail_ESS) is a diagnostic for sampling efficiency of the tails of the posterior(see Bürkner, 2017; Vehtari *et al.*, 2017). Parameters under the "Population-Level" heading indicate fitness function parameters estimated across the entire dataset. Parameters under the "Group Level" heading indicate variation (standard deviation) in annual mean deviations from the population level parameters. The different parameters estimated are the fitness intercept a, and the selection differential b. The suffixes indicate the treatment and nestling rank level that a parameter was estimated at relative to the first-hatched food supplemented nestling group. For example, the selection differential on mass for a non-food supplemented first-hatched nestling would be sum of *b_First-hatched_Fed* and *b_Treatmentunfed*. If there is no suffix for a parameter the same parameter value was estimated for all food treatment and rank groupings.

**Age 5 Model of Selection**							
**Fluctuating Linear Mass by Group, Total observations = 4093**							
Group-Level	Effects:						
~Year	(Number	of	levels:	24)			
	Estimate	Est.Error	l-95% CI	u-95%CI	Rhat	Bulk_ESS	Tail_ESS
sd(a_First-hatched_Fed)	0.25	0.16	0.01	0.61	1	1293	1755
sd(a_Second-hatched)	0.52	0.21	0.11	0.95	1	1050	988
sd(a_Treatmentunfed)	0.42	0.21	0.04	0.85	1	1019	892
sd(a_Second-hatched:Treatmentunfed)	0.25	0.19	0.01	0.72	1	1925	1860
sd(b_First-hatched_Fed)	0.21	0.15	0.01	0.55	1	1219	1647
sd(b_Second-hatched)	0.26	0.19	0.01	0.69	1	1443	1858
sd(b_Treatmentunfed)	0.26	0.18	0.01	0.66	1	1006	1782
sd(b_ Second-hatched:Treatmentunfed)	0.35	0.26	0.01	0.97	1	1176	1419
Population-Level	Effects:						
	Estimate	Est.Error	l-95% CI	u-95%CI	Rhat	Bulk_ESS	Tail_ESS
a_First-hatched_Fed	3.75	0.22	3.35	4.22	1	2689	2078
a_Second-hatched	-0.92	0.29	-1.49	-0.34	1	2233	2500
a_Treatmentunfed	-0.08	0.27	-0.64	0.46	1	2136	2316
a_ Second-hatched:Treatmentunfed	-1.08	0.33	-1.72	-0.42	1	2329	2221
b_First-hatched_Fed	1.43	0.17	1.08	1.76	1	2114	1935
b_ Second-hatched	-0.09	0.23	-0.55	0.35	1	2633	2467
b_Treatmentunfed	0.11	0.21	-0.31	0.52	1	2457	2446
b_ Second-hatched:Treatmentunfed	-0.22	0.28	-0.75	0.34	1	3064	2467

Supplementary Table 12: Top fitness function for survival from age 10 to age 15 based on ΔLOOIC comparisons. For each model parameter the mean (Estimate), standard deviation (Est. Error), lower 95% credible interval (l-95% CI), and upper 95% credible interval (u-95% CI) are reported for the posterior distribution of that parameter. Rhat is an indicate of convergence of chains and if it is considerably greater than 1 the chains have not converged. The Bulk effective sampling size (Bulk_ESS) is a diagnostic for sampling efficiency of the bulk of the posterior and the tail effective sampling size (Tail_ESS) is a diagnostic for sampling efficiency of the tails of the posterior(see Bürkner, 2017; Vehtari *et al.*, 2017). Parameters under the "Population-Level" heading indicate fitness function parameters estimated across the entire dataset. Parameters under the "Group Level" heading indicate variation (standard deviation) in annual mean deviations from the population level parameters. The different parameters estimated are the fitness intercept a, and the selection gradient b. "_Mass" or "_Wing" following the parameter indicate the estimate for mass or wing length, respectively. The suffixes indicate the treatment and nestling rank level that a parameter was estimated at relative to the first-hatched food supplemented nestling group. For example, the selection differential on mass for a non-food supplemented first-hatched nestling would be sum of *b_First-hatched_Fed* and *b_Treatmentunfed*. If there is no suffix for a parameter, the same parameter value was estimated for all food treatment and rank groupings.

**Age 10 Model of Selection**							
**Synchronous Fluctuating Bivariate Wing & Mass, Total observations = 3577**							
Group-Level	Effects:						
~Year	(Number	of	levels:	24)			
	Estimate	Est.Error	l-95% CI	u-95%CI	Rhat	Bulk_ESS	Tail_ESS
sd(a)	0.39	0.2	0.02	0.82	1	560	553
sd(b_Wing)	0.11	0.08	0.01	0.31	1	638	591
sd(b_Mass)	0.18	0.12	0.01	0.45	1.01	393	586
Population-Level	Effects:						
	Estimate	Est.Error	l-95% CI	u-95%CI	Rhat	Bulk_ESS	Tail_ESS
a_First-hatched_Fed	4.46	0.28	3.94	5.02	1.01	582	520
a_ Second-hatched	0.09	0.41	-0.66	0.88	1	609	468
a_Treatmentunfed	-0.5	0.32	-1.13	0.1	1	632	484
a_ Second-hatched:Treatmentunfed	-0.17	0.48	-1.07	0.75	1	679	618
b_Wing_First-hatched_Fed	-0.24	0.37	-0.96	0.55	1	589	588
b_ Wing_ Second-hatched	-0.62	0.48	-1.58	0.27	1	667	546
b_ Wing_Treatmentunfed	0.04	0.38	-0.71	0.78	1	674	545
b_ Wing_ Second-hatched:Treatmentunfed	0.03	0.51	-0.9	1.05	1	708	540
b_Mass_First-hatched_Fed	1.32	0.33	0.68	1.94	1	562	590
b_ Mass_ Second-hatched	0.52	0.42	-0.27	1.32	1	622	590
b_ Mass_Treatmentunfed	0.09	0.35	-0.59	0.75	1	607	445
b_ Mass_ Second-hatched:Treatmentunfed	0.6	0.47	-0.3	1.58	1	678	627

Supplementary Table 13: Top fitness function for survival from age 15 to age 20 based on ΔLOOIC comparisons. For each model parameter the mean (Estimate), standard deviation (Est. Error), lower 95% credible interval (l-95% CI), and upper 95% credible interval (u-95% CI) are reported for the posterior distribution of that parameter. Rhat is an indicate of convergence of chains and if it is considerably greater than 1 the chains have not converged. The Bulk effective sampling size (Bulk_ESS) is a diagnostic for sampling efficiency of the bulk of the posterior and the tail effective sampling size (Tail_ESS) is a diagnostic for sampling efficiency of the tails of the posterior(see Bürkner, 2017; Vehtari *et al.*, 2017). Parameters under the "Population-Level" heading indicate fitness function parameters estimated across the entire dataset. Parameters under the "Group Level" heading indicate variation (standard deviation) in annual mean deviations from the population level parameters. The different parameters estimated are the fitness intercept a, and the selection gradient b. "_Mass" or "_Wing" following the parameter indicate the estimate for mass or wing length, respectively. The suffixes indicate the treatment and nestling rank level that a parameter was estimated at relative to the first-hatched food supplemented nestling group. For example, the selection differential on mass for a non-food supplemented first-hatched nestling would be sum of *b_First-hatched_Fed* and *b_Treatmentunfed*. If there is no suffix for a parameter, the same parameter value was estimated for all food treatment and rank groupings.

**Age 15 Model of Selection**							
**Synchronous Fluctuating Bivariate Wing & Mass, Total observations = 3511**							
Group-Level	Effects:						
~Year	(Number	of	levels:	24)			
	Estimate	Est.Error	l-95% CI	u-95%CI	Rhat	Bulk_ESS	Tail_ESS
sd(a)	0.43	0.23	0.03	0.92	1	666	580
sd(b_Wing)	0.24	0.16	0.02	0.57	1	719	590
sd(b_Mass)	0.36	0.17	0.04	0.74	1	432	529
Population-Level	Effects:						
	Estimate	Est.Error	l-95% CI	u-95%CI	Rhat	Bulk_ESS	Tail_ESS
a_First-hatched_Fed	4.38	0.26	3.89	4.93	1	562	629
a_ Second-hatched	0.05	0.41	-0.73	0.83	1	513	464
a_Treatmentunfed	-0.07	0.33	-0.68	0.55	1	535	589
a_ Second-hatched:Treatmentunfed	0.37	0.53	-0.59	1.52	1	472	603
b_Wing_First-hatched_Fed	-0.12	0.3	-0.7	0.46	1	541	550
b_Wing_ Second-hatched	-0.2	0.37	-0.89	0.56	1	632	522
b_Wing_Treatmentunfed	0.39	0.35	-0.29	1.1	1	452	570
b_Wing Second-hatched:Treatmentunfed	-1.06	0.49	-2.03	-0.15	1	711	632
b_Mass _First-hatched_Fed	0.58	0.28	0	1.13	1	648	517
b_Mass _ Second-hatched	1.09	0.38	0.36	1.82	1	529	497
b_Mass _Treatmentunfed	0.69	0.34	0.05	1.38	1	606	560
b_Mass_ Second-hatched:Treatmentunfed	0.33	0.48	-0.62	1.22	1	659	621

Supplementary Table 14: Top fitness function for survival from age 20 to age 25 based on ΔLOOIC comparisons. For each model parameter the mean (Estimate), standard deviation (Est. Error), lower 95% credible interval (l-95% CI), and upper 95% credible interval (u-95% CI) are reported for the posterior distribution of that parameter. Rhat is an indicate of convergence of chains and if it is considerably greater than 1 the chains have not converged. The Bulk effective sampling size (Bulk_ESS) is a diagnostic for sampling efficiency of the bulk of the posterior and the tail effective sampling size (Tail_ESS) is a diagnostic for sampling efficiency of the tails of the posterior(see Bürkner, 2017; Vehtari *et al.*, 2017). Parameters under the "Population-Level" heading indicate fitness function parameters estimated across the entire dataset. Parameters under the "Group Level" heading indicate variation (standard deviation) in annual mean deviations from the population level parameters. The different parameters estimated are the fitness intercept a, and the selection differential b. The suffixes indicate the treatment and nestling rank level that a parameter was estimated at relative to the first-hatched food supplemented nestling group. For example, the selection differential on mass for a non-food supplemented first-hatched nestling would be sum of *b_First-hatched_Fed* and *b_Treatmentunfed*. If there is no suffix for a parameter, the same parameter value was estimated for all food treatment and rank groupings.

**Age 20 Model of Selection**							
**Synchronous Fluctuating Linear Mass, Total observations = 3336**							
Group-Level	Effects:						
~Year	(Number	of	levels:	24)			
	Estimate	Est.Error	l-95% CI	u-95%CI	Rhat	Bulk_ESS	Tail_ESS
sd(a)	0.47	0.23	0.07	0.93	1	600	633
sd(b)	0.17	0.13	0.01	0.48	1	632	626
Population-Level	Effects:						
	Estimate	Est.Error	l-95% CI	u-95%CI	Rhat	Bulk_ESS	Tail_ESS
a_First-hatched_Fed	4.08	0.27	3.56	4.59	1	553	590
a_ Second-hatched	0.18	0.37	-0.53	0.97	1	616	414
a_Treatmentunfed	0.48	0.32	-0.18	1.13	1	579	444
a_ Second-hatched:Treatmentunfed	-0.2	0.54	-1.26	0.89	1	590	586
b_First-hatched_Fed	0.88	0.17	0.56	1.16	1	680	617
b_ Second-hatched	-0.23	0.31	-0.83	0.38	1	638	441
b_Treatmentunfed	0.11	0.19	-0.26	0.5	1	671	530
b _ Second-hatched:Treatmentunfed	0.78	0.37	0.1	1.55	1	651	629

Supplementary Table 15: Top fitness function for survival from age 25 to age 30 based on ΔLOOIC comparisons. For each model parameter the mean (Estimate), standard deviation (Est. Error), lower 95% credible interval (l-95% CI), and upper 95% credible interval (u-95% CI) are reported for the posterior distribution of that parameter. Rhat is an indicate of convergence of chains and if it is considerably greater than 1 the chains have not converged. The Bulk effective sampling size (Bulk_ESS) is a diagnostic for sampling efficiency of the bulk of the posterior and the tail effective sampling size (Tail_ESS) is a diagnostic for sampling efficiency of the tails of the posterior(see Bürkner, 2017; Vehtari *et al.*, 2017). Parameters under the "Population-Level" heading indicate fitness function parameters estimated across the entire dataset. Parameters under the "Group Level" heading indicate variation (standard deviation) in annual mean deviations from the population level parameters. The different parameters estimated are the fitness intercept a, and the selection differential b. The suffixes indicate the treatment and nestling rank level that a parameter was estimated at relative to the first-hatched food supplemented nestling group. For example, the selection differential on mass for a non-food supplemented first-hatched nestling would be sum of *b_First-hatched_Fed* and *b_Treatmentunfed*. If there is no suffix for a parameter, the same parameter value was estimated for all food treatment and rank groupings.

**Age 25 Model of Selection**							
**Heterogenous Fluctuating Linear Mass, Total observations = 3306**							
Group-Level	Effects:						
~Year	(Number	of	levels:	24)			
	Estimate	Est.Error	l-95% CI	u-95%CI	Rhat	Bulk_ESS	Tail_ESS
sd(a_First-hatched_Fed)	0.36	0.27	0.01	0.99	1	554	631
sd(a_ Second-hatched)	0.7	0.53	0.02	1.92	1	671	494
sd(a_Treatmentunfed)	0.4	0.3	0.02	1.09	1.01	621	504
sd(a_ Second-hatched:Treatmentunfed)	1.35	0.86	0.1	3.3	1	565	628
sd(b_First-hatched_Fed)	0.31	0.23	0.01	0.84	1	501	590
sd(b_ Second-hatched)	0.9	0.49	0.06	1.99	1	444	495
sd(b_Treatmentunfed)	0.27	0.22	0.01	0.82	1	616	583
sd(b_ Second-hatched:Treatmentunfed)	1.03	0.7	0.07	2.58	1	565	535
Population-Level	Effects:						
	Estimate	Est.Error	l-95% CI	u-95%CI	Rhat	Bulk_ESS	Tail_ESS
a_First-hatched_Fed	4.49	0.29	3.95	5.09	1	677	639
a_ Second-hatched	0.94	0.59	-0.11	2.27	1	559	591
a_Treatmentunfed	0.44	0.37	-0.3	1.17	1	536	551
a_ Second-hatched:Treatmentunfed	0.74	0.79	-0.7	2.28	1	623	506
b_First-hatched_Fed	0.57	0.24	0.07	1.04	1	596	516
b_ Second-hatched	0.17	0.44	-0.67	1.01	1	737	564
b_Treatmentunfed	0.44	0.29	-0.1	1.06	1	686	580
b_ Second-hatched:Treatmentunfed	0.17	0.58	-0.9	1.35	1	742	455

Supplementary Table 16: Top fitness function for survival from age 30 to age 35 based on ΔLOOIC comparisons. For each model parameter the mean (Estimate), standard deviation (Est. Error), lower 95% credible interval (l-95% CI), and upper 95% credible interval (u-95% CI) are reported for the posterior distribution of that parameter. Rhat is an indicate of convergence of chains and if it is considerably greater than 1 the chains have not converged. The Bulk effective sampling size (Bulk_ESS) is a diagnostic for sampling efficiency of the bulk of the posterior and the tail effective sampling size (Tail_ESS) is a diagnostic for sampling efficiency of the tails of the posterior(see Bürkner, 2017; Vehtari *et al.*, 2017). Parameters under the "Population-Level" heading indicate fitness function parameters estimated across the entire dataset. Parameters under the "Group Level" heading indicate variation (standard deviation) in annual mean deviations from the population level parameters. The different parameters estimated are the fitness intercept a, and the selection differential b. The suffixes indicate the treatment and nestling rank level that a parameter was estimated at relative to the first-hatched food supplemented nestling group. For example, the selection differential on mass for a non-food supplemented first-hatched nestling would be sum of *b_First-hatched_Fed* and *b_Treatmentunfed*. If there is no suffix for a parameter, the same parameter value was estimated for all food treatment and rank groupings.

**Age 30 Model of Selection**							
**Synchronous Fluctuating Linear Mass, Total observations = 3028**							
Group-Level	Effects:						
~Year	(Number	of	levels:	24)			
	Estimate	Est.Error	l-95% CI	u-95%CI	Rhat	Bulk_ESS	Tail_ESS
sd(a)	0.53	0.27	0.08	1.14	1	569	627
sd(b)	0.25	0.15	0.02	0.6	1	708	633
Population-Level	Effects:						
	Estimate	Est.Error	l-95% CI	u-95%CI	Rhat	Bulk_ESS	Tail_ESS
a_First-hatched_Fed	4.51	0.34	3.88	5.24	1.01	516	535
a_ Second-hatched	0.3	0.49	-0.63	1.2	1	628	520
a_Treatmentunfed	0.28	0.37	-0.39	0.98	1	647	635
a_ Second-hatched:Treatmentunfed	0.61	0.69	-0.71	2.01	1.01	632	525
b_First-hatched_Fed	0.56	0.24	0.11	1.02	1.01	568	488
b_ Second-hatched	-0.34	0.44	-1.16	0.52	1.01	522	444
b_Treatmentunfed	0.48	0.27	-0.07	1.01	1.01	619	552
b_ Second-hatched:Treatmentunfed	0.86	0.51	-0.18	1.85	1	654	516

Supplementary Table 17: Top fitness function for survival from age 35 to age 40 based on ΔLOOIC comparisons. For each model parameter the mean (Estimate), standard deviation (Est. Error), lower 95% credible interval (l-95% CI), and upper 95% credible interval (u-95% CI) are reported for the posterior distribution of that parameter. Rhat is an indicate of convergence of chains and if it is considerably greater than 1 the chains have not converged. The Bulk effective sampling size (Bulk_ESS) is a diagnostic for sampling efficiency of the bulk of the posterior and the tail effective sampling size (Tail_ESS) is a diagnostic for sampling efficiency of the tails of the posterior(see Bürkner, 2017; Vehtari *et al.*, 2017). Parameters under the "Population-Level" heading indicate fitness function parameters estimated across the entire dataset. Parameters under the "Group Level" heading indicate variation (standard deviation) in annual mean deviations from the population level parameters. The different parameters estimated are the fitness intercept a, and the selection differential b. The suffixes indicate the treatment and nestling rank level that a parameter was estimated at relative to the first-hatched food supplemented nestling group. For example, the selection differential on mass for a non-food supplemented first-hatched nestling would be sum of *b_First-hatched_Fed* and *b_Treatmentunfed*. If there is no suffix for a parameter, the same parameter value was estimated for all food treatment and rank groupings

**Age 35 Model of Selection**							
**Synchronous Fluctuating Linear Mass, Total observations = 2855**							
Group-Level	Effects:						
~Year	(Number	of	levels:	24)			
	Estimate	Est.Error	l-95% CI	u-95%CI	Rhat	Bulk_ESS	Tail_ESS
sd(a)	0.32	0.27	0.01	1	1	638	602
sd(b)	0.55	0.25	0.11	1.13	1	662	572
Population-Level	Effects:						
	Estimate	Est.Error	l-95% CI	u-95%CI	Rhat	Bulk_ESS	Tail_ESS
a_First-hatched_Fed	4.68	0.31	4.12	5.25	1	594	568
a_ Second-hatched	0.76	0.55	-0.27	1.89	1.01	874	548
a_Treatmentunfed	1.1	0.48	0.2	2.02	1	616	591
a_ Second-hatched:Treatmentunfed	-0.21	0.78	-1.72	1.28	1	582	458
b_First-hatched_Fed	0.52	0.31	-0.12	1.11	1	665	576
b_ Second-hatched	0.05	0.49	-0.93	0.97	1	611	574
b_Treatmentunfed	0.29	0.37	-0.44	0.96	1	613	547
b_ Second-hatched:Treatmentunfed	-0.8	0.65	-2.12	0.47	1	586	590

Supplementary Table 18: Top fitness function for survival from age 40 to age fledging based on ΔLOOIC comparisons. For each model parameter the mean (Estimate), standard deviation (Est. Error), lower 95% credible interval (l-95% CI), and upper 95% credible interval (u-95% CI) are reported for the posterior distribution of that parameter. Rhat is an indicate of convergence of chains and if it is considerably greater than 1 the chains have not converged. The Bulk effective sampling size (Bulk_ESS) is a diagnostic for sampling efficiency of the bulk of the posterior and the tail effective sampling size (Tail_ESS) is a diagnostic for sampling efficiency of the tails of the posterior(see Bürkner, 2017; Vehtari *et al.*, 2017). Parameters under the "Population-Level" heading indicate fitness function parameters estimated across the entire dataset. Parameters under the "Group Level" heading indicate variation (standard deviation) in annual mean deviations from the population level parameters. The different parameters estimated are the fitness intercept a, and the selection differential b. The suffixes indicate the treatment and nestling rank level that a parameter was estimated at relative to the first-hatched food supplemented nestling group. For example, the selection differential on mass for a non-food supplemented first-hatched nestling would be sum of *b_First-hatched_Fed* and *b_Treatmentunfed*. If there is no suffix for a parameter, the same parameter value was estimated for all food treatment and rank groupings

**Age 40 Model of Selection**							
**Heterogenous Fluctuating Linear Mass, Total observations = 1912**							
Group-Level	Effects:						
~Year	(Number	of	levels:	24)			
	Estimate	Est.Error	l-95% CI	u-95%CI	Rhat	Bulk_ESS	Tail_ESS
sd(a_First-hatched_Fed)	0.48	0.37	0.02	1.33	1	687	444
sd(a_ Second-hatched)	0.89	0.65	0.03	2.4	1	626	552
sd(a_Treatmentunfed)	0.8	0.6	0.03	2.23	1	559	591
sd(a_ Second-hatched:Treatmentunfed)	2.02	1.44	0.1	5.4	1	574	516
sd(b_First-hatched_Fed)	0.72	0.33	0.1	1.38	1	592	628
sd(b_ Second-hatched)	1.02	0.63	0.08	2.34	1.01	614	543
sd(b_Treatmentunfed)	0.53	0.4	0.02	1.49	1	707	682
sd(b_ Second-hatched:Treatmentunfed)	1.43	0.95	0.07	3.43	1	607	594
Population-Level	Effects:						
	Estimate	Est.Error	l-95% CI	u-95%CI	Rhat	Bulk_ESS	Tail_ESS
a_First-hatched_Fed	4.74	0.38	4.05	5.48	1	601	579
a_ Second-hatched	0.3	0.62	-0.76	1.6	1	662	586
a_Treatmentunfed	0.8	0.57	-0.26	1.98	1	494	514
a_ Second-hatched:Treatmentunfed	0.54	0.84	-1.01	2.3	1	585	631
b_First-hatched_Fed	-0.22	0.34	-0.94	0.44	1	600	534
b_ Second-hatched	0.15	0.48	-0.86	1.04	1.01	580	553
b_Treatmentunfed	0.29	0.41	-0.5	1.05	1	491	583
b_ Second-hatched:Treatmentunfed	-0.37	0.71	-1.72	1.03	1	590	552

Supplementary Table 19: Among year standard deviation in fitness function parameters by selective period. Values in brackets indicate the 95% credible intervals for each estimate. Estimates are from the best model (lowest LOOIC) for each selective period. Estimates for a linear model of fitness are displayed for age 0 for comparison to all other selective periods and an estimate of selection gradient on wing length are included if the best (lowest LOOIC) or a top (ΔLOOIC) fitness model included wing length (Ages 0, 5, 10, 15, 20, 35).

**Parameter**			**Age**		
	**0**	**5**	**10**	**15**	**20**
**SD(β_0)**	0.57 [0.40, 0.81]	0.58 [0.37, 0.87]	0.39 [0.02, 0.82]	0.43 [0.03, 0.92]	0.47 [0.07, 0.93]
**SD(β_{Mass})**	0.10 [0, 0.26]	0.19 [0.01, 0.42]	0.11 [0.01, 0.31]	0.36 [0.04, 0.74]	0.17 [0.01, 0.48]
**SD(β_{Wing})**	0.13 [0.01, 0.30]	0.15 [0.01, 0.36] *	0.11 [0.01, 0.45]	0.24 [0.02, 0.57]	0.14 [0, 0.39] *
**SD(θ_{Mass})**	0.47 [0.12, 0.84]	-	-	-	-
**SD(θ_{Wing})**	3.1 [1.8, 4.9]	-	-	-	-
**SD(W_{max})**	0.64 [0.04, 1.6]	-	-	-	-

**Parameter**			**Age**	
	**25**	**30**	**35**	**40**
**SD(β_0)**	0.30 [0.01, 0.86]	0.53 [0.08, 1.14]	0.32 [0.01, 1.0]	0.42 [0.02, 1.04]
**SD(β_{Mass})**	0.41 [0.07, 0.81]	0.25 [0.02, 0.60]	0.55 [0.11, 1.0]	0.83 [0.37, 1.42]
**SD(β_{Wing})**	-	-	0.51 [0.03, 1.18] *	-

***** Estimate is from a top model (LOOIC <5), but this parameter is not part of the best model

Supplementary Table 20: ΔLOOIC values for fitness functions evaluated for survival from age 0 to age 5 as a function of mass and/or wing length. ΔLOOIC values are the differences between LOOIC values of the best model (lowest LOOIC value) and LOOIC value for a given model. Models with a ΔLOOIC <5 are bolded. Airwin, SSTWin, PDO, and lagged PDO correspond to models with air temperature, sea-surface temperature, Pacific Decadal Oscillation, and Pacific Decadal Oscillation two years prior as predictors of fitness function parameters. Synchronous indicates a model where the environmental variable effects selection on all nestling rank and food treatment group the same way and heterogenous indicates the effects of the environmental variable effects selection differently depending on the nestling rank and food treatment.

Survival Period	Model	ΔLOOIC
Survival from 0 to 5 Days	**Synchronous AirWin**	**0**
	**Heterogenous AirWin**	**1.3**
	Annual Fluctuations Only	15
	Synchronous SSTWin	16.5
	Heterogenous SSTWin	17.9
	Heterogenous Lagged PDO	25.9
	Heterogenous PDO	25.9
	Synchronous Lagged PDO	28.1
	Synchronous PDO	43.9

Supplementary Table 21: ΔLOOIC values for fitness functions evaluated for survival from age 5 to age 10 as a function of mass and/or wing length. ΔLOOIC values are the differences between LOOIC values of the best model (lowest LOOIC value) and LOOIC value for a given model. Models with a ΔLOOIC <5 are bolded. Airwin, SSTWin, PDO, and lagged PDO correspond to models with air temperature, sea-surface temperature, Pacific Decadal Oscillation, and Pacific Decadal Oscillation two years prior as predictors of fitness function parameters. Synchronous indicates a model where the environmental variable effects selection on all nestling rank and food treatment group the same way and heterogenous indicates the effects of the environmental variable effects selection differently depending on the nestling rank and food treatment.

Survival Period	Model	ΔLOOIC
Survival from 5 to 10 Days	**Annual Fluctuations Only**	**0**
	**Heterogenous PDO**	**0.8**
	**Synchronous AirWin**	**0.9**
	**Synchronous PDO**	**0.9**
	**Synchronous Lagged PDO**	**1.8**
	**Synchronous SSTWin**	**3.2**
	**Heterogenous AirWin**	**4.4**
	Heterogenous Lagged PDO	8.5
	Heterogenous SSTWin	Did not Converge

Supplementary Table 22: ΔLOOIC values for fitness functions evaluated for survival from age 10 to age 15 as a function of mass and/or wing length. ΔLOOIC values are the differences between LOOIC values of the best model (lowest LOOIC value) and LOOIC value for a given model. Models with a ΔLOOIC <5 are bolded. Airwin, SSTWin, PDO, and lagged PDO correspond to models with air temperature, sea-surface temperature, Pacific Decadal Oscillation, and Pacific Decadal Oscillation two years prior as predictors of fitness function parameters. Synchronous indicates a model where the environmental variable effects selection on all nestling rank and food treatment group the same way and heterogenous indicates the effects of the environmental variable effects selection differently depending on the nestling rank and food treatment.

Survival Period	Model	ΔLOOIC
Survival from 10 to 15 Days	**Synchronous AirWin**	**0**
	**Synchronous SSTWin**	**2.9**
	**Annual Fluctuations Only**	**4.8**
	Synchronous Lagged PDO	6.5
	Heterogenous Lagged PDO	6.7
	Synchronous PDO	7.1
	Heterogenous AirWin	7.1
	Heterogenous PDO	16.3
	Heterogenous SSTWin	Did not Converge

Supplementary Table 23: ΔLOOIC values for fitness functions evaluated for survival from age 15 to age 20 as a function of mass and/or wing length. ΔLOOIC values are the differences between LOOIC values of the best model (lowest LOOIC value) and LOOIC value for a given model. Models with a ΔLOOIC <5 are bolded. Airwin, SSTWin, PDO, and lagged PDO correspond to models with air temperature, sea-surface temperature, Pacific Decadal Oscillation, and Pacific Decadal Oscillation two years prior as predictors of fitness function parameters. Synchronous indicates a model where the environmental variable effects selection on all nestling rank and food treatment group the same way and heterogenous indicates the effects of the environmental variable effects selection differently depending on the nestling rank and food treatment.

Survival Period	Model	ΔLOOIC
Survival from 15 to 20 Days	Synchronous AirWin	0
	Heterogenous PDO	19.5
	Annual Fluctuations Only	5.4
	Synchronous SSTWin	9.4
	Synchronous PDO	9.5
	Synchronous Lagged PDO	9.5
	Heterogenous AirWin	9.7
	Heterogenous Lagged PDO	10.4
	Heterogenous SSTWin	Did not converge

Supplementary Table 24: ΔLOOIC values for fitness functions evaluated for survival from age 20 to age 25 as a function of mass and/or wing length. ΔLOOIC values are the differences between LOOIC values of the best model (lowest LOOIC value) and LOOIC value for a given model. Models with a ΔLOOIC <5 are bolded. Airwin, SSTWin, PDO, and lagged PDO correspond to models with air temperature, sea-surface temperature, Pacific Decadal Oscillation, and Pacific Decadal Oscillation two years prior as predictors of fitness function parameters. Synchronous indicates a model where the environmental variable effects selection on all nestling rank and food treatment group the same way and heterogenous indicates the effects of the environmental variable effects selection differently depending on the nestling rank and food treatment.

Survival Period	Model	ΔLOOIC
Survival from 20 to 25 Days	**Synchronous PDO**	**0**
	**Annual Fluctuations Only**	**2.9**
	**Synchronous Lagged PDO**	**3.9**
	**Synchronous SSTWin**	**4.8**
	Synchronous AirWin	5.8
	Heterogenous PDO	6.2
	Heterogenous AirWin	7.7
	Heterogenous SSTWin	9.8
	Heterogenous Lagged PDO	11.5

Supplementary Table 25: ΔLOOIC values for fitness functions evaluated for survival from age 25 to age 30 as a function of mass and/or wing length. ΔLOOIC values are the differences between LOOIC values of the best model (lowest LOOIC value) and LOOIC value for a given model. Models with a ΔLOOIC <5 are bolded. Airwin, SSTWin, PDO, and lagged PDO correspond to models with air temperature, sea-surface temperature, Pacific Decadal Oscillation, and Pacific Decadal Oscillation two years prior as predictors of fitness function parameters. Synchronous indicates a model where the environmental variable effects selection on all nestling rank and food treatment group the same way and heterogenous indicates the effects of the environmental variable effects selection differently depending on the nestling rank and food treatment.

Survival Period	Model	ΔLOOIC
Survival from 25 to 30 Days	Heterogenous Lagged PDO	0
	Heterogenous AirWin	1.1
	Synchronous SSTWin	1.6
	Annual Fluctuations Only	2.1
	Synchronous PDO	2.9
	Synchronous Lagged PDO	4.5
	Synchronous AirWin	4.6
	Heterogenous PDO	7.3
	Heterogenous SSTWin	Did not converge

Supplementary Table 26: ΔLOOIC values for fitness functions evaluated for survival from age 30 to age 35 as a function of mass and/or wing length. ΔLOOIC values are the differences between LOOIC values of the best model (lowest LOOIC value) and LOOIC value for a given model. Models with a ΔLOOIC <5 are bolded. Airwin, SSTWin, PDO, and lagged PDO correspond to models with air temperature, sea-surface temperature, Pacific Decadal Oscillation, and Pacific Decadal Oscillation two years prior as predictors of fitness function parameters. Synchronous indicates a model where the environmental variable effects selection on all nestling rank and food treatment group the same way and heterogenous indicates the effects of the environmental variable effects selection differently depending on the nestling rank and food treatment.

Survival Period	Model	ΔLOOIC
Survival from 30 to 35 Days	Synchronous SSTWin	0
	Synchronous PDO	0.5
	Annual Fluctuations Only	1
	Synchronous AirWin	1.3
	Heterogenous Lagged PDO	2
	Synchronous Lagged PDO	2.2
	Heterogenous PDO	4.3
	Heterogenous AirWin	5.6
	Heterogenous SSTWin	6.6

Supplementary Table 27: ΔLOOIC values for fitness functions evaluated for survival from age 35 to age 40 as a function of mass and/or wing length. ΔLOOIC values are the differences between LOOIC values of the best model (lowest LOOIC value) and LOOIC value for a given model. Models with a ΔLOOIC <5 are bolded. Airwin, SSTWin, PDO, and lagged PDO correspond to models with air temperature, sea-surface temperature, Pacific Decadal Oscillation, and Pacific Decadal Oscillation two years prior as predictors of fitness function parameters. Synchronous indicates a model where the environmental variable effects selection on all nestling rank and food treatment group the same way and heterogenous indicates the effects of the environmental variable effects selection differently depending on the nestling rank and food treatment.

Survival Period	Model	ΔLOOIC
Survival from 35 to 40 Days	Annual Fluctuations Only	0
	Synchronous AirWin	0.9
	Synchronous SSTWin	1.3
	Heterogenous Lagged PDO	1.8
	Synchronous Lagged PDO	2.3
	Synchronous PDO	3.2
	Heterogenous PDO	3.5
	Heterogenous AirWin	5.1
	Heterogenous SSTWin	Did not converge

Supplementary Table 28: ΔLOOIC values for fitness functions evaluated for survival from age 0 to age 5 as a function of mass and/or wing length. ΔLOOIC values are the differences between LOOIC values of the best model (lowest LOOIC value) and LOOIC value for a given model. Models with a ΔLOOIC <5 are bolded. Airwin, SSTWin, PDO, and lagged PDO correspond to models with air temperature, sea-surface temperature, Pacific Decadal Oscillation, and Pacific Decadal Oscillation two years prior as predictors of fitness function parameters. Synchronous indicates a model where the environmental variable effects selection on all nestling rank and food treatment group the same way and heterogenous indicates the effects of the environmental variable effects selection differently depending on the nestling rank and food treatment.

Survival Period	Model	ΔLOOIC
Survival from 40 to Fledge	Heterogenous PDO	0
	Heterogenous Lagged PDO	0.5
	Annual Fluctuations Only	1.1
	Synchronous Lagged PDO	2.5
	Synchronous AirWin	2.9
	Synchronous SSTWin	3.1
	Synchronous PDO	3.4
	Heterogenous AirWin	3.9
	Heterogenous SSTWin	Did not converge

Supplementary Table 29: Top fitness function for survival from age 0 to age 5 based on ΔLOOIC comparisons. For each model parameter the mean (Estimate), standard deviation (Est. Error), lower 95% credible interval (l-95% CI), and upper 95% credible interval (u-95% CI) are reported for the posterior distribution of that parameter. Rhat is an indicate of convergence of chains and if it is considerably greater than 1 the chains have not converged. The Bulk effective sampling size (Bulk_ESS) is a diagnostic for sampling efficiency of the bulk of the posterior and the tail effective sampling size (Tail_ESS) is a diagnostic for sampling efficiency of the tails of the posterior(see Bürkner, 2017; Vehtari *et al.*, 2017). Parameters under the "Population-Level" heading indicate fitness function parameters estimated across the entire dataset. Parameters under the "Group Level" heading indicate variation (standard deviation) in annual mean deviations from the population level parameters. The different parameters estimated are the maximum fitness Wmax, the trait specific optimum θ, the correlation between wing length and mass ρ, and the width of the fitness function for each trait ω. The suffices indicate the treatment and nestling rank level that a parameter was estimated at relative to the first-hatched food supplemented nestling group. For example, the optimal mass for a non-food supplemented first-hatched nestling would be sum of θ*_Mass_First-hatched_Fed* and θ*_ Mass_Treatmentunfed*. If there is no suffix for a parameter the same parameter was assumed for all food treatment and rank groupings. An "AirWin", "SSTWin", "pdo", or "pdo_lagged" suffix indicates the estimate of the linear relationship between a function parameter and our chosen air temperature, sea-surface temperature, Pacific Decadal Oscillation, or lagged Pacific Decadal Oscillation parameters.

**Age 0**<br>**Air Temp Synchronous**							
Group-Level	Effects:						
~Year	(Number	of	levels:	22)			
	Estimate	Est.Error	l-95% CI	u-95%CI	Rhat	Bulk_ESS	Tail_ESS
sd(Wmax)	0.68	0.5	0.03	1.85	1	4495	6236
sd(θ_Mass)	0.46	0.19	0.11	0.87	1	2566	3388
sd(θ_Wing)	3.6	1.05	1.89	5.96	1	3971	5176
Population-Level	Effects:						
	Estimate	Est.Error	l-95% CI	u-95%CI	Rhat	Bulk_ESS	Tail_ESS
Wmax	4.35	0.42	3.62	5.25	1	9749	8081
ρ	0.01	0.15	-0.29	0.3	1	6216	7630
θ_Mass_First-hatched_Fed	0.8	0.32	0.14	1.42	1	6281	7227
θ_AirWin	0.14	0.06	0.04	0.27	1	5177	5841
θ_Second-hatched	1.43	0.3	0.88	2.05	1	8631	8632
θ_Treatmentunfed	0.3	0.29	-0.23	0.89	1	5812	7086
θ_Second-hatched:Treatmentunfed	0.07	0.61	-1.17	1.23	1	5234	6043
θ_Wing_First-hatched_Fed	4.55	1.6	1.83	8.16	1	5051	5399
θ_Wing_AirWin	-0.93	0.32	-1.63	-0.41	1	4911	6089
θ_Wing_Second-hatched	-1.48	1.76	-5.1	1.92	1	7841	8027
θ_Wing_Treatmentunfed	-3.48	1.19	-6.09	-1.39	1	6922	6659
θ_Wing_ Second-hatched:Treatmentunfed	-6.82	2.74	-12.73	-2	1	6237	6369
ω_Mass	5.04	0.47	4.23	6.07	1	8896	7224
ω_Wing	16.48	3.31	10.98	23.89	1	4794	6169

Supplementary Table 30: Top fitness function for survival from age 5 to age 10 based on ΔLOOIC comparisons. For each model parameter the mean (Estimate), standard deviation (Est. Error), lower 95% credible interval (l-95% CI), and upper 95% credible interval (u-95% CI) are reported for the posterior distribution of that parameter. Rhat is an indicate of convergence of chains and if it is considerably greater than 1 the chains have not converged. The Bulk effective sampling size (Bulk_ESS) is a diagnostic for sampling efficiency of the bulk of the posterior and the tail effective sampling size (Tail_ESS) is a diagnostic for sampling efficiency of the tails of the posterior(see Bürkner, 2017; Vehtari *et al.*, 2017). Parameters under the "Population-Level" heading indicate fitness function parameters estimated across the entire dataset. Parameters under the "Group Level" heading indicate variation (standard deviation) in annual mean deviations from the population level parameters. The different parameters estimated are the fitness intercept a, and the selection differential b. The suffixes indicate the treatment and nestling rank level that a parameter was estimated at relative to the first-hatched food supplemented nestling group. For example, the selection differential on mass for a non-food supplemented first-hatched nestling would be sum of *b_First-hatched_Fed* and *b_Treatmentunfed*. If there is no suffix for a parameter the same parameter value was estimated for all food treatment and rank groupings. A "AirWin", "SSTWin", "pdo", or "pdo_lagged" suffix indicates the estimate of the linear relationship between a function parameter and our chosen air temperature, sea-surface temperature, Pacific Decadal Oscillation, or lagged Pacific Decadal Oscillation parameters.

**Age 5**							
**Annual Variation Only**							
Group-Level	Effects:						
~Year	(Number	of	levels :	22)			
	Estimate	Est.Error	l-95% CI	u-95%CI	Rhat	Bulk_ESS	Tail_ESS
sd(a)	0.6	0.14	0.38	0.91	1	4548	7005
sd(b)	0.19	0.12	0.01	0.44	1	2636	3715
Population-Level	Effects:						
	Estimate	Est.Error	l-95% CI	u-95%CI	Rhat	Bulk_ESS	Tail_ESS
a_First-hatched_Fed	3.75	0.25	3.28	4.24	1	4819	7537
a_ Second-hatched	-1.06	0.27	-1.6	-0.53	1	5479	7631
a_Treatmentunfed	-0.27	0.25	-0.77	0.22	1	5482	7285
a_ Second-hatched:Treatmentunfed	-0.85	0.33	-1.48	-0.21	1	5366	7462
b_First-hatched_Fed	1.45	0.17	1.1	1.79	1	5720	7497
b_ Second-hatched	-0.12	0.22	-0.55	0.33	1	5568	7545
b_Treatmentunfed	0	0.2	-0.4	0.4	1	5563	7434
b_ Second-hatched:Treatmentunfed	-0.14	0.28	-0.68	0.4	1	5480	8164

Supplementary Table 31: Top fitness function for survival from age 10 to age 15 based on ΔLOOIC comparisons. For each model parameter the mean (Estimate), standard deviation (Est. Error), lower 95% credible interval (l-95% CI), and upper 95% credible interval (u-95% CI) are reported for the posterior distribution of that parameter. Rhat is an indicate of convergence of chains and if it is considerably greater than 1 the chains have not converged. The Bulk effective sampling size (Bulk_ESS) is a diagnostic for sampling efficiency of the bulk of the posterior and the tail effective sampling size (Tail_ESS) is a diagnostic for sampling efficiency of the tails of the posterior(see Bürkner, 2017; Vehtari *et al.*, 2017). Parameters under the "Population-Level" heading indicate fitness function parameters estimated across the entire dataset. Parameters under the "Group Level" heading indicate variation (standard deviation) in annual mean deviations from the population level parameters. The different parameters estimated are the fitness intercept a, and the selection gradient b. "_Mass" or "_Wing" following the parameter indicate the estimate for mass or wing length, respectively. The suffixes indicate the treatment and nestling rank level that a parameter was estimated at relative to the first-hatched food supplemented nestling group. For example, the selection gradeintl on mass for a non-food supplemented first-hatched nestling would be sum of *b_Mass_First-hatched_Fed* and *b*_Mass_*Treatmentunfed*. If there is no suffix for a parameter, the same parameter value was estimated for all food treatment and rank groupings. A "AirWin", "SSTWin", "pdo", or "pdo_lagged" suffix indicates the estimate of the linear relationship between a function parameter and our chosen air temperature, sea-surface temperature, Pacific Decadal Oscillation, or lagged Pacific Decadal Oscillation parameters.

**Age 10**							
**Air Temperature Synchronous**							
Group-Level	Effects:						
~Year	(Number	of	levels:	22)			
	Estimate	Est.Error	l-95% CI	u-95%CI	Rhat	Bulk_ESS	Tail_ESS
sd(a)	0.51	0.25	0.06	1.03	1	3307	3710
sd(b_Wing)	0.15	0.12	0.01	0.44	1	5827	6910
sd(b_Mass)	0.2	0.14	0.01	0.54	1	3631	5899
Population-Level	Effects:						
	Estimate	Est.Error	l-95% CI	u-95%CI	Rhat	Bulk_ESS	Tail_ESS
a_First-hatched_Fed	4.4	0.33	3.76	5.07	1	10416	8827
a_ Second-hatched	0.1	0.43	-0.71	0.95	1	11755	9942
a_Treatmentunfed	-0.28	0.33	-0.93	0.37	1	12051	9722
a_AirWin	0.05	0.06	-0.06	0.17	1	9512	8431
a_ Second-hatched:Treatmentunfed	-0.12	0.53	-1.14	0.91	1	11821	9876
b_ Wing _First-hatched_Fed	-0.33	0.38	-1.07	0.44	1	7748	7884
b_ Wing _Second-hatched	-0.53	0.48	-1.47	0.42	1	9698	8594
b_ Wing _Treatmentunfed	0	0.38	-0.74	0.76	1	7810	7929
b_ Wing _AirWin	0.01	0.06	-0.1	0.12	1	12519	8960
b_ Wing _Second-hatched:Treatmentunfed	0.07	0.54	-0.98	1.13	1	9770	9156
b_Mass_First-hatched_Fed	1.08	0.35	0.4	1.75	1	8128	8636
b_Mass_Second-hatched	0.15	0.45	-0.73	1.02	1	9952	8923
b_Mass_Treatmentunfed	0.15	0.36	-0.55	0.84	1	8504	8116
b_Mass_AirWin	0.12	0.05	0.02	0.22	1	11659	8865
b_Mass_Second-hatched:Treatmentunfed	0.81	0.52	-0.18	1.86	1	10027	8785

Supplementary Table 32: Top fitness function for survival from age 15 to age 20 based on ΔLOOIC comparisons. For each model parameter the mean (Estimate), standard deviation (Est. Error), lower 95% credible interval (l-95% CI), and upper 95% credible interval (u-95% CI) are reported for the posterior distribution of that parameter. Rhat is an indicate of convergence of chains and if it is considerably greater than 1 the chains have not converged. The Bulk effective sampling size (Bulk_ESS) is a diagnostic for sampling efficiency of the bulk of the posterior and the tail effective sampling size (Tail_ESS) is a diagnostic for sampling efficiency of the tails of the posterior(see Bürkner, 2017; Vehtari *et al.*, 2017). Parameters under the "Population-Level" heading indicate fitness function parameters estimated across the entire dataset. Parameters under the "Group Level" heading indicate variation (standard deviation) in annual mean deviations from the population level parameters. The different parameters estimated are the fitness intercept a, and the selection gradient b. "_Mass" or "_Wing" following the parameter indicate the estimate for mass or wing length, respectively. The suffixes indicate the treatment and nestling rank level that a parameter was estimated at relative to the first-hatched food supplemented nestling group. For example, the selection gradient on mass for a non-food supplemented first-hatched nestling would be sum of *b_Mass_First-hatched_Fed* and *b*_Mass_*Treatmentunfed*. If there is no suffix for a parameter, the same parameter value was estimated for all food treatment and rank groupings. A "AirWin", "SSTWin", "pdo", or "pdo_lagged" suffix indicates the estimate of the linear relationship between a function parameter and our chosen air temperature, sea-surface temperature, Pacific Decadal Oscillation, or lagged Pacific Decadal Oscillation parameters.

**Age 15**							
**Air Temperature Synchronous**							
Group-Level	Effects:						
~Year	(Number	of	levels:	22)			
	Estimate	Est.Error	l-95% CI	u-95%CI	Rhat	Bulk_ESS	Tail_ESS
sd(a)	0.44	0.27	0.03	1.05	1	3448	6276
sd(b)	0.27	0.17	0.01	0.65	1	3870	6156
sd(b_Mass)	0.36	0.19	0.03	0.76	1	3273	4671
Population-Level	Effects:						
	Estimate	Est.Error	l-95% CI	u-95%CI	Rhat	Bulk_ESS	Tail_ESS
a_First-hatched_Fed	4.75	0.39	4	5.53	1	10949	9156
a_ Second-hatched	0.22	0.43	-0.59	1.08	1	15791	10032
a_Treatmentunfed	0	0.34	-0.67	0.67	1	17369	10185
a_AirWin	-0.12	0.08	-0.29	0.05	1	9362	8230
a_ Second-hatched:Treatmentunfed	0.62	0.62	-0.56	1.87	1	15285	9732
b_Wing_First-hatched_Fed	0.48	0.34	-0.19	1.15	1	9386	8963
b_ Wing_Second-hatched	0.05	0.38	-0.7	0.81	1	11794	9674
b_Treatmentunfed	0.42	0.33	-0.23	1.08	1	11045	8795
b _ Wing_AirWin	-0.19	0.07	-0.33	-0.06	1	10053	9411
b _ Wing_ Second-hatched:Treatmentunfed	-0.99	0.51	-2	0.02	1	12842	9638
b _Mass _First-hatched_Fed	0.33	0.31	-0.26	0.95	1	10189	9335
b _Mass _ Second-hatched	0.95	0.42	0.12	1.77	1	11180	8974
b _Mass _Treatmentunfed	0.56	0.33	-0.08	1.21	1	10962	9458
b _Mass _AirWin	0.1	0.06	-0.02	0.21	1	8849	7504
b _Mass _ Second-hatched:Treatmentunfed	0.44	0.51	-0.56	1.45	1	11200	9384

Supplementary Table 33: Top fitness function for survival from age 20 to age 25 based on ΔLOOIC comparisons. For each model parameter the mean (Estimate), standard deviation (Est. Error), lower 95% credible interval (l-95% CI), and upper 95% credible interval (u-95% CI) are reported for the posterior distribution of that parameter. Rhat is an indicate of convergence of chains and if it is considerably greater than 1 the chains have not converged. The Bulk effective sampling size (Bulk_ESS) is a diagnostic for sampling efficiency of the bulk of the posterior and the tail effective sampling size (Tail_ESS) is a diagnostic for sampling efficiency of the tails of the posterior(see Bürkner, 2017; Vehtari *et al.*, 2017). Parameters under the "Population-Level" heading indicate fitness function parameters estimated across the entire dataset. Parameters under the "Group Level" heading indicate variation (standard deviation) in annual mean deviations from the population level parameters. The different parameters estimated are the fitness intercept a, and the selection gradient b. "_Mass" or "_Wing" following the parameter indicate the estimate for mass or wing length, respectively. The suffixes indicate the treatment and nestling rank level that a parameter was estimated at relative to the first-hatched food supplemented nestling group. For example, the selection differential on mass for a non-food supplemented first-hatched nestling would be sum of *b_First-hatched_Fed* and *b_Treatmentunfed*. If there is no suffix for a parameter, the same parameter value was estimated for all food treatment and rank groupings. A "AirWin", "SSTWin", "pdo", or "pdo_lagged" suffix indicates the estimate of the linear relationship between a function parameter and our chosen air temperature, sea-surface temperature, Pacific Decadal Oscillation, or lagged Pacific Decadal Oscillation parameters.

**Age 20**							
**PDO Synchronous**							
Group-Level	Effects:						
~Year	(Number	of	levels:	22)			
	Estimate	Est.Error	l-95% CI	u-95%CI	Rhat	Bulk_ESS	Tail_ESS
sd(a)	0.34	0.21	0.02	0.82	1	4444	5995
sd(b)	0.17	0.14	0.01	0.51	1	4623	6454
Population-Level	Effects:						
	Estimate	Est.Error	l-95% CI	u-95%CI	Rhat	Bulk_ESS	Tail_ESS
a_First-hatched_Fed	4.03	0.29	3.48	4.63	1	11918	8393
a_ Second-hatched	0.22	0.42	-0.57	1.06	1	14453	10050
a_Treatmentunfed	0.56	0.36	-0.12	1.27	1	12989	9603
a_pdo	-0.16	0.18	-0.52	0.2	1	13562	9270
a_ Second-hatched:Treatmentunfed	-0.14	0.59	-1.28	1.06	1	14361	9810
b _First-hatched_Fed	1.03	0.19	0.66	1.38	1	10624	8690
b _ Second-hatched	-0.18	0.33	-0.85	0.46	1	12871	9512
b _Treatmentunfed	0.05	0.23	-0.4	0.49	1	12030	8973
b _pdo	0.18	0.11	-0.04	0.41	1	13070	9450
b _ Second-hatched:Treatmentunfed	0.65	0.41	-0.16	1.45	1	12821	9356

Supplementary Table 34: Top fitness function for survival from age 25 to age 30 based on ΔLOOIC comparisons. For each model parameter the mean (Estimate), standard deviation (Est. Error), lower 95% credible interval (l-95% CI), and upper 95% credible interval (u-95% CI) are reported for the posterior distribution of that parameter. Rhat is an indicate of convergence of chains and if it is considerably greater than 1 the chains have not converged. The Bulk effective sampling size (Bulk_ESS) is a diagnostic for sampling efficiency of the bulk of the posterior and the tail effective sampling size (Tail_ESS) is a diagnostic for sampling efficiency of the tails of the posterior(see Bürkner, 2017; Vehtari *et al.*, 2017). Parameters under the "Population-Level" heading indicate fitness function parameters estimated across the entire dataset. Parameters under the "Group Level" heading indicate variation (standard deviation) in annual mean deviations from the population level parameters. The different parameters estimated are the fitness intercept a, and the selection differential b. The suffixes indicate the treatment and nestling rank level that a parameter was estimated at relative to the first-hatched food supplemented nestling group. For example, the selection differential on mass for a non-food supplemented first-hatched nestling would be sum of *b_First-hatched_Fed* and *b_Treatmentunfed*. If there is no suffix for a parameter the same parameter value was estimated for all food treatment and rank groupings. A "AirWin", "SSTWin", "pdo", or "pdo_lagged" suffix indicates the estimate of the linear relationship between a function parameter and our chosen air temperature, sea-surface temperature, Pacific Decadal Oscillation, or lagged Pacific Decadal Oscillation parameters.

**Age 25**							
**Lagged PDO Heterogenous**							
Group-Level	Effects:						
~Year	(Number	of	levels:	22)			
	Estimate	Est.Error	l-95% CI	u-95%CI	Rhat	Bulk_ESS	Tail_ESS
sd(a)	0.38	0.27	0.02	1.01	1	3801	4564
sd(b)	0.43	0.21	0.05	0.88	1	2527	3005
Population-Level	Effects:						
	Estimate	Est.Error	l-95% CI	u-95%CI	Rhat	Bulk_ESS	Tail_ESS
a_First-hatched_Fed	4.44	0.32	3.85	5.08	1	7675	7925
a_ Second-hatched	0.7	0.56	-0.34	1.84	1	9855	8443
a_Treatmentunfed	0.6	0.45	-0.25	1.51	1	8236	8252
a_pdo_lagged	-0.52	0.3	-1.12	0.06	1	7315	7778
a_ Second-hatched:Treatmentunfed	1	0.79	-0.57	2.52	1	9905	9133
a_ Second-hatched:pdo_lagged	-0.01	0.49	-0.98	0.94	1	8303	8736
a_Treatmentunfed:pdo_lagged	0.71	0.37	0.01	1.45	1	7731	8704
a_ Second-hatched:Treatmentunfed:pdo_lagged	0.9	0.59	-0.25	2.07	1	7926	8010
b _First-hatched_Fed	0.38	0.29	-0.2	0.94	1	6613	7969
b _ Second-hatched	0.53	0.39	-0.22	1.28	1	6367	8054
b _Treatmentunfed	0.68	0.34	0.02	1.35	1	6096	8085
b _pdolagged	0	0.28	-0.54	0.57	1	6380	8118
b _ Second-hatched:Treatmentunfed	0.16	0.48	-0.78	1.13	1	6754	7898
b _ Second-hatched:pdo_lagged	0.51	0.4	-0.27	1.3	1	6629	8057
b _Treatmentunfed:pdo_lagged	0.3	0.31	-0.32	0.88	1	6298	8058
b _ Second-hatched:Treatmentunfed:pdo_lagged	-0.43	0.47	-1.35	0.48	1	6095	7294

Supplementary Table 35: Top fitness function for survival from age 30 to age 35 based on ΔLOOIC comparisons. For each model parameter the mean (Estimate), standard deviation (Est. Error), lower 95% credible interval (l-95% CI), and upper 95% credible interval (u-95% CI) are reported for the posterior distribution of that parameter. Rhat is an indicate of convergence of chains and if it is considerably greater than 1 the chains have not converged. The Bulk effective sampling size (Bulk_ESS) is a diagnostic for sampling efficiency of the bulk of the posterior and the tail effective sampling size (Tail_ESS) is a diagnostic for sampling efficiency of the tails of the posterior(see Bürkner, 2017; Vehtari *et al.*, 2017). Parameters under the "Population-Level" heading indicate fitness function parameters estimated across the entire dataset. Parameters under the "Group Level" heading indicate variation (standard deviation) in annual mean deviations from the population level parameters. The different parameters estimated are the fitness intercept a, and the selection differential b. The suffixes indicate the treatment and nestling rank level that a parameter was estimated at relative to the first-hatched food supplemented nestling group. For example, the selection differential on mass for a non-food supplemented first-hatched nestling would be sum of *b_First-hatched_Fed* and *b_Treatmentunfed*. If there is no suffix for a parameter the same parameter value was estimated for all food treatment and rank groupings. A "AirWin", "SSTWin", "pdo", or "pdo_lagged" suffix indicates the estimate of the linear relationship between a function parameter and our chosen air temperature, sea-surface temperature, Pacific Decadal Oscillation, or lagged Pacific Decadal Oscillation parameters.

**Age 30**							
**SST Synchronous**							
Group-Level	Effects:						
~Year	(Number	of	levels:	22)			
	Estimate	Est.Error	l-95% CI	u-95%CI	Rhat	Bulk_ESS	Tail_ESS
sd(a)	0.54	0.3	0.04	1.19	1	3868	4189
sd(b)	0.23	0.16	0.01	0.62	1	3773	5996
Population-Level	Effects:						
	Estimate	Est.Error	l-95% CI	u-95%CI	Rhat	Bulk_ESS	Tail_ESS
a_First-hatched_Fed	0.98	0.87	-0.68	2.71	1	10638	9274
a_ Second-hatched	-0.01	0.5	-0.97	0.99	1	18865	9670
a_Treatmentunfed	0.19	0.41	-0.62	0.97	1	16119	8248
a_SSTWin	0.39	0.09	0.21	0.56	1	10687	9177
a_ Second-hatched:Treatmentunfed	0.84	0.68	-0.46	2.19	1	17594	9564
b _First-hatched_Fed	-0.77	0.7	-2.14	0.58	1	10911	9691
b _ Second-hatched	-0.37	0.46	-1.28	0.54	1	12921	9711
b _Treatmentunfed	0.45	0.28	-0.09	1.02	1	16080	9956
b _SSTWin	0.15	0.07	0.02	0.28	1	10834	9153
b _ Second-hatched:Treatmentunfed	0.85	0.52	-0.16	1.86	1	11923	9406

Supplementary Table 36: Top fitness function for survival from age 35 to age 40 based on ΔLOOIC comparisons. For each model parameter the mean (Estimate), standard deviation (Est. Error), lower 95% credible interval (l-95% CI), and upper 95% credible interval (u-95% CI) are reported for the posterior distribution of that parameter. Rhat is an indicate of convergence of chains and if it is considerably greater than 1 the chains have not converged. The Bulk effective sampling size (Bulk_ESS) is a diagnostic for sampling efficiency of the bulk of the posterior and the tail effective sampling size (Tail_ESS) is a diagnostic for sampling efficiency of the tails of the posterior(see Bürkner, 2017; Vehtari *et al.*, 2017). Parameters under the "Population-Level" heading indicate fitness function parameters estimated across the entire dataset. Parameters under the "Group Level" heading indicate variation (standard deviation) in annual mean deviations from the population level parameters. The different parameters estimated are the fitness intercept a, and the selection differential b. The suffixes indicate the treatment and nestling rank level that a parameter was estimated at relative to the first-hatched food supplemented nestling group. For example, the selection differential on mass for a non-food supplemented first-hatched nestling would be sum of *b_First-hatched_Fed* and *b_Treatmentunfed*. If there is no suffix for a parameter the same parameter value was estimated for all food treatment and rank groupings. A "AirWin", "SSTWin", "pdo", or "pdo_lagged" suffix indicates the estimate of the linear relationship between a function parameter and our chosen air temperature, sea-surface temperature, Pacific Decadal Oscillation, or lagged Pacific Decadal Oscillation parameters.

**Age 35**<br>**Annual Fluctuations**							
Group-Level	Effects:						
~Year	(Number	of	levels:	22)			
	Estimate	Est.Error	l-95% CI	u-95%CI	Rhat	Bulk_ESS	Tail_ESS
sd(a)	0.33	0.26	0.01	0.99	1	5487	5417
sd(b)	0.55	0.25	0.11	1.1	1	4149	3332
Population-Level	Effects:						
	Estimate	Est.Error	l-95% CI	u-95%CI	Rhat	Bulk_ESS	Tail_ESS
a_First-hatched_Fed	4.65	0.32	4.04	5.31	1	10431	7944
a_ Second-hatched	0.77	0.55	-0.27	1.91	1	12397	9109
a_Treatmentunfed	1.08	0.48	0.18	2.05	1	10986	9229
a_ Second-hatched:Treatmentunfed	-0.2	0.78	-1.71	1.38	1	12789	8949
b _First-hatched_Fed	0.53	0.32	-0.1	1.14	1	8036	8314
b _ Second-hatched	0.04	0.48	-0.93	0.96	1	10275	9252
b _Treatmentunfed	0.3	0.37	-0.43	1.02	1	7826	8187
b _ Second-hatched:Treatmentunfed	-0.85	0.66	-2.16	0.41	1	10098	8978

Supplementary Table 37: Top fitness function for survival from age 40 to fledging based on ΔLOOIC comparisons. For each model parameter the mean (Estimate), standard deviation (Est. Error), lower 95% credible interval (l-95% CI), and upper 95% credible interval (u-95% CI) are reported for the posterior distribution of that parameter. Rhat is an indicate of convergence of chains and if it is considerably greater than 1 the chains have not converged. The Bulk effective sampling size (Bulk_ESS) is a diagnostic for sampling efficiency of the bulk of the posterior and the tail effective sampling size (Tail_ESS) is a diagnostic for sampling efficiency of the tails of the posterior(see Bürkner, 2017; Vehtari *et al.*, 2017). Parameters under the "Population-Level" heading indicate fitness function parameters estimated across the entire dataset. Parameters under the "Group Level" heading indicate variation (standard deviation) in annual mean deviations from the population level parameters. The different parameters estimated are the fitness intercept a, and the selection differential b. The suffixes indicate the treatment and nestling rank level that a parameter was estimated at relative to the first-hatched food supplemented nestling group. For example, the selection differential on mass for a non-food supplemented first-hatched nestling would be sum of *b_First-hatched_Fed* and *b_Treatmentunfed*. If there is no suffix for a parameter the same parameter value was estimated for all food treatment and rank groupings. A "AirWin", "SSTWin", "pdo", or "pdo_lagged" suffix indicates the estimate of the linear relationship between a function parameter and our chosen air temperature, sea-surface temperature, Pacific Decadal Oscillation, or lagged Pacific Decadal Oscillation parameters.

**Age 40<br>PDO Synchronous**							
Group-Level	Effects:						
~Year	(Number	of	levels:	22)			
	Estimate	Est.Error	l-95% CI	u-95%CI	Rhat	Bulk_ESS	Tail_ESS
sd(a)	0.53	0.39	0.02	1.46	1	3617	6071
sd(b)	0.82	0.29	0.3	1.46	1	4468	3742
Population-Level	Effects:						
	Estimate	Est.Error	l-95% CI	u-95%CI	Rhat	Bulk_ESS	Tail_ESS
a_First-hatched_Fed	4.57	0.38	3.87	5.35	1	10367	8428
a_ Second-hatched	-0.23	0.46	-1.12	0.68	1	12939	8996
a_Treatmentunfed	0.45	0.48	-0.46	1.42	1	13226	9714
a_pdo	-0.26	0.26	-0.8	0.22	1	8583	5808
a_ Second-hatched:Treatmentunfed	0.4	0.75	-1.01	1.91	1	14249	9498
b _First-hatched_Fed	-0.1	0.36	-0.79	0.63	1	8358	8503
b _ Second-hatched	0.25	0.4	-0.52	1.04	1	11437	9723
b _Treatmentunfed	0.32	0.38	-0.42	1.08	1	10130	9353
b_pdo	0.19	0.24	-0.29	0.64	1	7826	8208
b_ Second-hatched:Treatmentunfed	0.21	0.57	-0.88	1.33	1	10676	9147

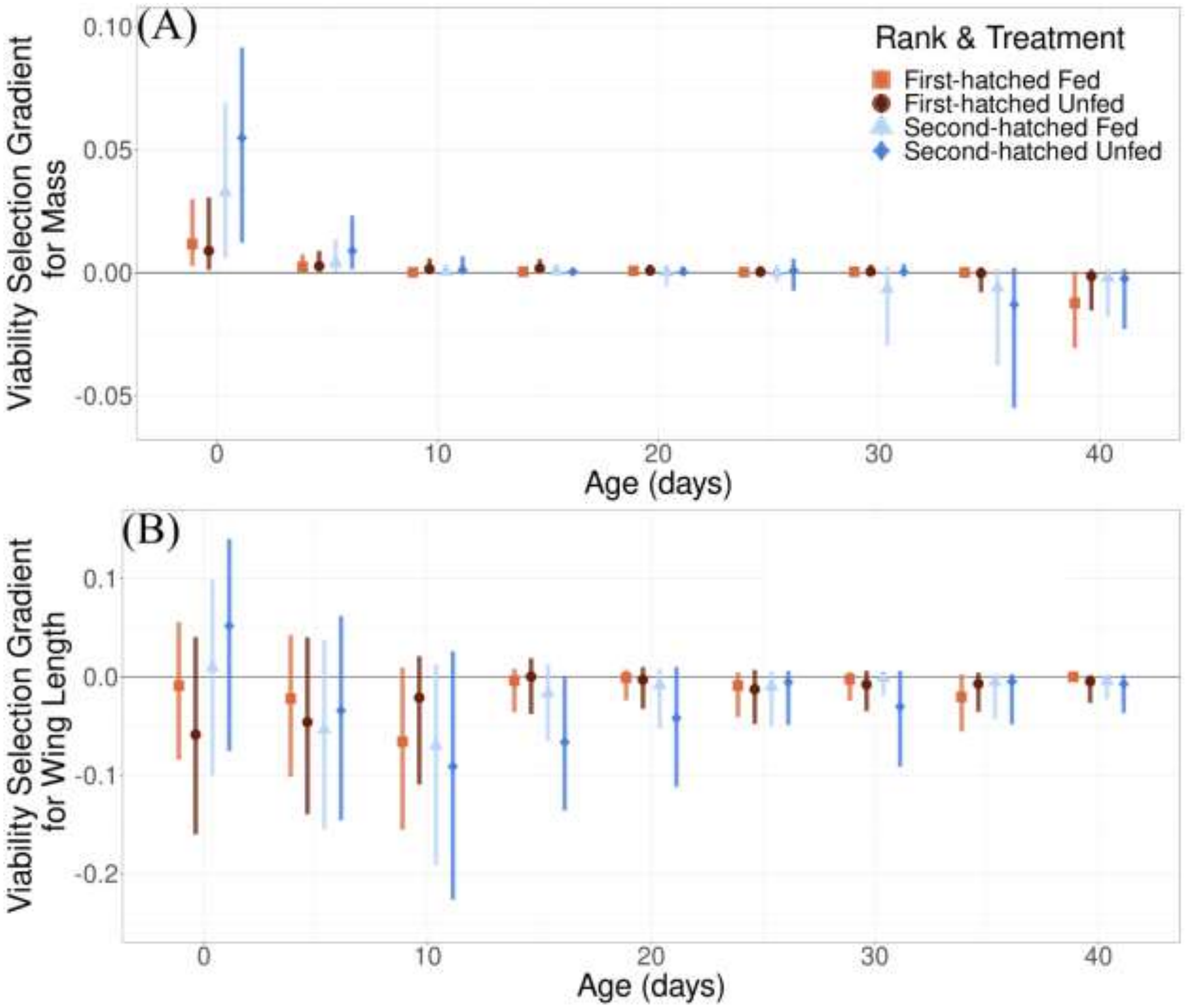

Supplemental Figure 1: Viability selection gradients for absolute A) mass and B) wing length. Selection gradients are transformed from logistic regression to the data scale following Janzen and Stern 1998 and Villemereuil et al. 2020. Points and 95% confidence intervals are coloured and grouped by nestling rank and food treatment groups. Brown colours indicate first hatched nestlings and blue colours indicate second hatched nestlings. Lighter colours indicate food supplemented nestlings while darker colours indicate non-food supplemented nestlings.

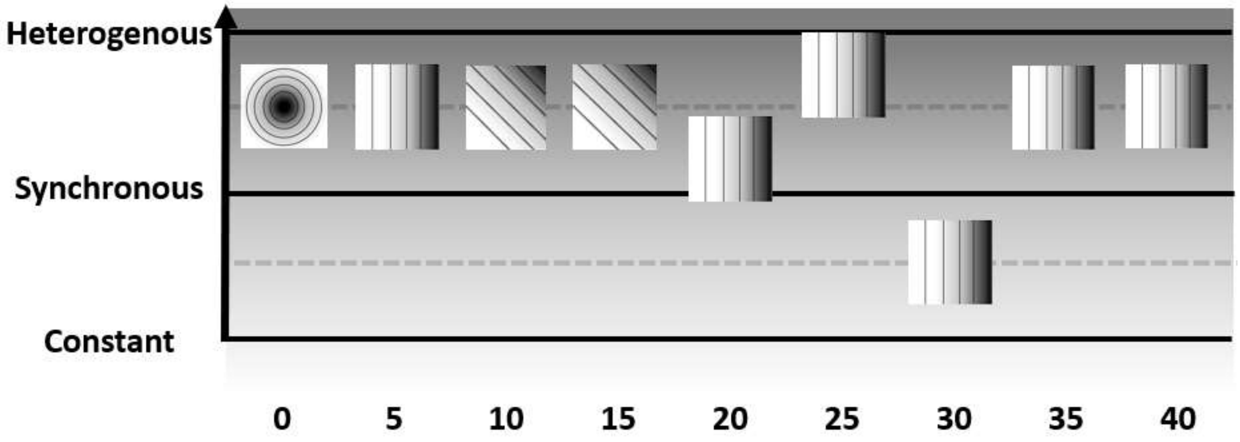

Supplemental Figure 2: Summary of selection functions and variability of selection parameters for each selective period (Age in number of days on the X-axis). On the Y-axis the "constant" indicates no annual variation in selection parameters, the "synchronous" that selection parameters varied similarly for all nestling rank and food treatment groups, and the "heterogenous" that each nestling rank and group varied differentially. For each selective period, a representative function is plotted indicating the best fitness function (based on LOOIC). Concentric circles on a square indicate a bivariate Gaussian function, diagonal lines on a square indicate a bivariate plane, and vertical lines on a plane indicate a linear function of mass. Position along the y-axis roughly corresponds to the proportion of top models that included a particular variance structure. For example, the best model for survival from age 5 to 10 was a linear model of mass and 50% of the top models included group specific fluctuations in selection.

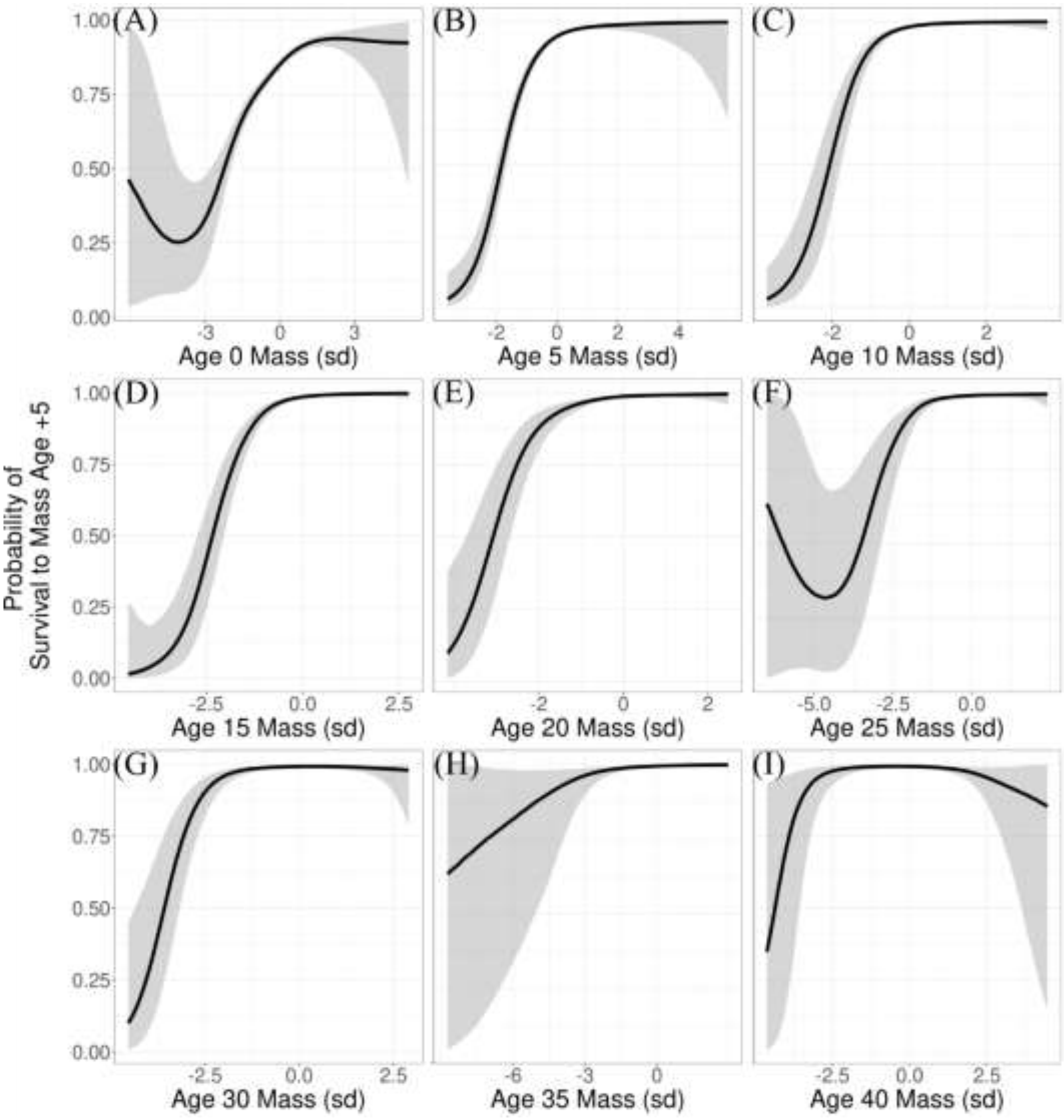

Supplemental Figure 3: Generalized additive model predicted survival as a function of nestling age specific mass. Plots A, B, C, D, E, F, G, H, & I correspond to survival to age 5, 10, 15, 20, 25, 30, 35, 40, & fledging. For each age class survival functions with associated 95% credible intervals (shaded areas) are displayed.

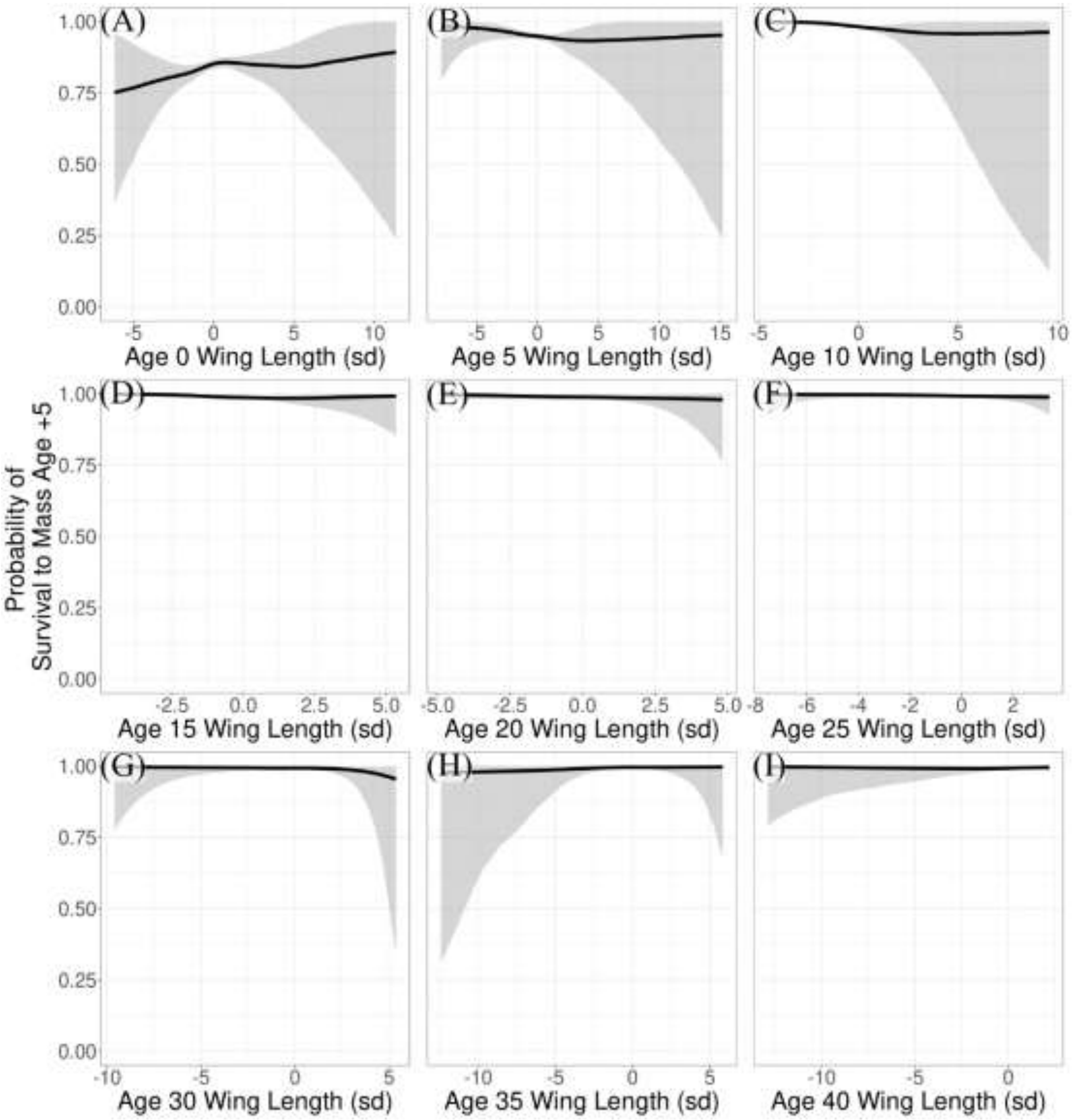

Supplemental Figure 4: Generalized additive model predicted survival as a function of nestling age specific wing length. Plots A, B, C, D, E, F, G, H, & I correspond to survival to age 5, 10, 15, 20, 25, 30, 35, 40, & fledging. For each age class survival functions with associated 95% credible intervals (shaded areas) are displayed.

References

Bürkner, P.-C. (2017) brms: An R Package for Bayesian Multilevel Models Using Stan. *Journal of Statistical Software*, **80**, 1–28.

Vehtari, A., Gelman, A. & Gabry, J. (2017) Practical Bayesian model evaluation using leave-one-out cross-validation and WAIC. *Statistics and Computing*, **27**, 1413–1432.

Villemereuil, P. de, Charmantier, A., Arlt, D., Bize, P., Brekke, P., Brouwer, L., *et al.* (2020) Fluctuating optimum and temporally variable selection on breeding date in birds and mammals. *Proceedings of the National Academy of Sciences*, **117**, 31969–31978.
